# Preconception and Early-Pregnancy Alcohol Consumption in Women and Men, Infertility and Risk for Miscarriage

**DOI:** 10.3390/nu18182953

**Published:** 2026-09-09

**Authors:** Morena Martinez Mayans, Celine H. X. Lin, Aline J. Boxem, Charlotte P. Van Brienen, Annemarie G. M. G. J. Mulders, Romy Gaillard, Vincent W. V. Jaddoe

**Affiliations:** 1 The Generation R Study Group, Erasmus MC, University Medical Center, 3000 CA Rotterdam, The Netherlands; m.martinezmayans@erasmusmc.nl (M.M.M.); c.lin@erasmusmc.nl (C.H.X.L.); a.j.boxem@erasmusmc.nl (A.J.B.); c.vanbrienen@erasmusmc.nl (C.P.V.B.); r.gaillard@erasmusmc.nl (R.G.); 2 Department of Pediatrics (Sp15-41), Sophia’s Children’s Hospital, Erasmus MC, University Medical Center, P.O. Box 2040, 3000 CA Rotterdam, The Netherlands; 3 Department of Obstetrics and Gynecology, Sophia’s Children’s Hospital, Erasmus MC, University Medical Center, 3000 CA Rotterdam, The Netherlands; a.mulders@erasmusmc.nl

**Keywords:** preconception, early pregnancy, alcohol, parental, infertility, miscarriage

## Abstract

**Background:** Alcohol consumption is common among women and men of reproductive age. The effects of alcohol use in the preconception and early-pregnancy periods on early reproductive outcomes are not well-known. **Methods:** In a population-based cohort study from preconception onwards among 3225 women and their partners, we examined the associations of preconception and early-pregnancy alcohol consumption, assessed by questionnaires, with fecundability (per-cycle probability of conceiving), infertility (time to pregnancy >12 months or use of assisted reproductive technology), and miscarriage (pregnancy loss before 22 weeks of gestation). **Results:** As compared to no alcohol consumption, preconception intake of <1 glass/week and ≥1 glass/week among women was associated with lower odds of infertility (odds ratio (OR) 0.61, 95% confidence interval (CI) 0.39, 0.94 and OR 0.54, 95% CI 0.35, 0.84, respectively). As compared to no alcohol consumption, early-pregnancy alcohol consumption among women of <1 glass/week and ≥1 glass/week was associated with increased odds of miscarriage (OR 1.99, 95% CI 1.36, 2.92 and OR 2.37, 95%CI 1.58, 3.57). Early-pregnancy binge drinking among women was also associated with increased odds of miscarriage (OR 1.52, 95%CI 1.04, 2.24). Alcohol consumption among men was not associated with infertility or miscarriage risk. **Conclusions:** The associations of low-to-moderate preconception alcohol consumption with lower odds of infertility might reflect healthier lifestyles, socioeconomic factors, and residual confounding by health-related abstinence from alcohol, all of which are associated with greater fertility. Therefore, preconception and early-pregnancy counseling should mainly focus on the risk of miscarriage and fetal health outcomes.

## 1. Introduction

Excessive alcohol consumption during pregnancy is a well-established risk factor for infant behavioral impairments, along with abnormal facial features, referred to as fetal alcohol syndrome [1,2,3,4,5]. Also, mild to moderate alcohol consumption in preconception and early pregnancy may adversely affect maternal and offspring health [6,7,8,9,10]. Despite the evidence of the harmful effects of alcohol consumption, it is still highly prevalent among women and men of reproductive age [11]. In the Netherlands, approximately 50% of women report mild to moderate alcohol consumption just before or during early pregnancy [12], making alcohol consumption a common dietary and lifestyle pattern during the preconception and early pregnancy. Early pregnancy seems to be a critical exposure window for the effects of alcohol on embryonic, fetal, and childhood outcomes. Alcohol consumption may cause hormonal dysregulation, which impairs follicle development and endometrial receptivity in women, and may negatively affect sperm quality in men. Furthermore, alcohol is directly toxic to the embryo as it readily crosses the placenta [13,14,15]. However, the effects of mild-to-moderate alcohol consumption on fertility and early-pregnancy outcomes are not well-known [16,17,18]. Results from previous studies among women and men suggest that preconception alcohol consumption is associated with a longer time to pregnancy and lower fecundability [19,20,21,22]. In addition, preconception alcohol consumption of ≥12 g per day in both women and men was associated with a lower success rate in assisted reproductive technology (ART) [23]. Studies focused on the associations of low-to-moderate alcohol consumption during pregnancy among women with the risk of miscarriage show inconsistent results [10,24]. Furthermore, in men, less is known about the effects of low-to-moderate alcohol consumption.

We hypothesized that already mild to moderate periconceptional alcohol consumption among both women and men leads to a higher risk of infertility and miscarriage. In a population-based cohort study from preconception onwards among 3225 women and their partners, we examined the associations of alcohol consumption with fecundability and risks of infertility and miscarriage, taking into account various socio-demographic, lifestyle-related, and pregnancy-related factors.

## 2. Materials and Methods

### 2.1. Study Design

This study was embedded in the Generation R *Next* Study, a population-based prospective cohort study in Rotterdam, the Netherlands, from the preconception period onwards [25]. Women and their partners could participate if they were 18 years or older, actively trying to conceive, or pregnant and living in Rotterdam. Participants were enrolled from 9 August 2017 to 1 July 2021. Enrollment was intended for those in the preconception or early-pregnancy period but was permitted until delivery. The Erasmus MC Medical Ethics Committee approved the study protocol. Written informed consent was obtained from all participants. Findings were reported in accordance with the Strengthening the Reporting of Observational Studies in Epidemiology (STROBE) guideline [26].

Couples were eligible to participate multiple times, starting either during a distinct preconception period or during a (subsequent) pregnancy. The study included 4036 participant episodes among 3604 unique women, and 3577 pregnancies resulted from 3200 unique female participants. In total, 33.2% of inclusions occurred during the preconception period, 52.8% in the first trimester, and 71.3% of partners participated in the study. In this study, episodes with no information on preconceptional or early-pregnancy alcohol consumption (*n* = 711) were excluded. For the time-to-pregnancy analyses, we included women and their partners who enrolled preconceptionally with information on their time-to-pregnancy or, if they did not conceive, the duration of actively pursuing pregnancy. This resulted in 945 episodes from 944 unique women. For the miscarriage analyses, we included women and their partners with a recorded pregnancy who were included before 22 weeks of pregnancy. This resulted in 2465 pregnancy episodes from 2259 unique women (Supplementary Figure S1).

### 2.2. Preconception and Early-Pregnancy Alcohol Consumption in Women and Men

Information on alcohol consumption was obtained through questionnaires administered during the preconception period and the first trimester, using categories of none, <1 glass per week, 1–3 glasses per week, 4–6 glasses per week, 1–2 glasses per day, 3–4 glasses per day, and ≥5 glasses per day, without distinguishing among specific types of alcoholic beverages. One standard glass corresponds to approximately 10 g of ethanol in the Netherlands [27]. In the preconception period, women reported their own and their partner’s average alcohol intake during the past 12 months. Preconception alcohol intake among women and men was categorized as none, <1 glass per week, or ≥1 glass per week. Preconception binge drinking, defined as consuming ≥6 glasses of alcohol on one day, was categorized as no binge drinking, <1 time per month, and ≥1 time per month. Due to both the limited number of preconceptionally participating women and their low alcohol intake, further subdivision of the higher-intake category was not feasible. During early pregnancy, women and men were asked to complete their own questionnaires. Women were asked to report their average alcohol consumption during the previous three months, primarily reflecting early pregnancy and part of the preconception period. If these data were missing, alcohol consumption prior to conception was used, either from the three months before conception or, if unavailable, the average intake during the 12 months prior to conception. During early pregnancy, alcohol intake was categorized as none, <1 glass per week, or ≥1 glass per week, whereas binge drinking was categorized as yes, binge drinking, or no, binge drinking. During early pregnancy, men were asked to report their average alcohol consumption in the three months prior to conception. When this information was unavailable, data from the women’s questionnaires regarding their partners’ drinking habits were used. If this was also unavailable, their alcohol intake for the 12 months prior to conception, reported by the female partner, was used, assuming that the alcohol intake of the partner remains fairly stable over time. Among men, early-pregnancy alcohol intake was categorized as none; <1 glass per week; 1–6 glasses per week; or >6 glasses per week, whereas binge drinking was categorized as no; <1 time per month; >1 time per month but less than once per week; or ≥1 time per week. Binge drinking by the male partner was only reported by women.

### 2.3. Time to Pregnancy and Miscarriage

Mode of conception and time to pregnancy were evaluated using preconception and early-pregnancy questionnaires, as described previously [28]. Participants reported when they refrained from contraceptives and started trying to conceive, along with dates for intrauterine insemination or assisted reproductive technology procedures, such as in vitro fertilization (IVF), and intracytoplasmic sperm injection (ICSI). The obstetric caregiver provided the date of the first day of the last menstrual period. For conceiving women, time to pregnancy was defined as the interval between the start date of active pregnancy pursuit and the first day of the last menstrual period. If conception did not occur, the active pregnancy pursuit duration was determined using the start date of active pregnancy pursuit to either the last day of study participation or the study’s end date. Fecundability was defined as the probability of conceiving per 28-day cycle. Time to pregnancy was dichotomized into fertile (conception within 12 months) and infertile (attempting conception over 12 months or using ART) [29]. Spontaneous miscarriage was defined as pregnancy loss before 22 weeks of gestation with dates provided by the obstetric caregiver [30]. Gestational age at miscarriage was determined using the first day of the last menstrual period, the ultrasonography-based due date, the IUI date, or the embryo transfer day minus 14 days in cases of IVF or ICSI.

### 2.4. Covariates

We constructed a directed acyclic graph (DAG) to identify potential covariates (Figures S2 and S3). We used questionnaires during preconception and early pregnancy to collect information about age at intake, parity (primipara, multipara), previous miscarriage (yes, no), ethnicity (Dutch, non-Dutch), educational level (primary, secondary, higher education), smoking (yes, no), illicit drug use (yes, no), and body mass index (BMI). Ethnicity was determined by the country of birth of the participant and their parents. If one of the participant’s parents was born abroad, the participant was classified as having a non-Dutch ethnic origin.

### 2.5. Statistical Power

We performed a power analysis, showing that based on a minimum of 700 subjects, we can detect with a type I error of 5% and a type II error of 20% (power 80%) an odds ratio of 2.48 if 10% of all subjects have the relevant exposure and the 1-year incidence of the outcome of interest is 10% [31].

### 2.6. Statistical Analysis

First, baseline characteristics were stratified by alcohol consumption categories, and differences between groups were assessed using Independent Student *t*-tests, Mann–Whitney U tests, and χ2 tests for categorical variables. Second, a non-response analysis was conducted by comparing characteristics of women and men with and without information on time to pregnancy or miscarriage using Independent Student *t*-tests, Mann–Whitney U tests, and χ2 tests for categorical variables. Third, we analyzed the association of preconception alcohol consumption with fecundability among women and men using Cox proportional hazards models (R package survival, 3.8-6). The fecundability time-to-event variable was measured in months (28 days) from the start of pregnancy pursuit until conception. In cases where conception was not achieved, time-to-event was measured up to the last reported pregnancy attempt, defined as the earlier of the voluntary withdrawal date and the study end date, and right-censored. Women receiving assisted conception had an uncertain time to pregnancy and were therefore excluded from the fecundability analyses. The resulting hazard ratio (HR) from the Cox model corresponds to the fecundability ratio (FR) (Methods S1). We examined the associations of early-pregnancy alcohol consumption among women and men with time to miscarriage using Cox proportional hazards models (R package survival). The outcome was the occurrence or non-occurrence of a miscarriage. The time-to-event variable for miscarriage was based on gestational age in weeks, whereas stillbirth or live birth were censored at 22 weeks. Ectopic pregnancies and induced abortions were categorized as no miscarriage because of underlying pathophysiological differences and were treated as censoring events at the corresponding gestational age. For both models, we evaluated the proportional hazards assumption for the covariates through Schoenfeld residuals and assessed influence using deviance residuals. Fifth, we analyzed the associations of alcohol consumption with the odds of infertility and miscarriage among women and men through logistic regression models. We conducted 7 sensitivity analyses. First, since some women participated multiple times, we repeated the analyses using only their first study participation. Second, we excluded participants who underwent assisted conception to assess whether any association was affected by assisted reproductive technology. Third, to account for the right-skewed distribution of time-to-pregnancy, the Cox proportional hazards regression was repeated after excluding the top 5% of the distribution. Fourth, to account for potential confounding from partners’ alcohol consumption, we repeated the analyses with adjustment for partners’ alcohol consumption. Fifth, we repeated the time-to-pregnancy analyses with alcohol consumption in the past three months, to account for any changes in alcohol consumption patterns in the preconception period. Sixth, we repeated the analyses using <1 glass per week and <1 time per month as the reference groups to assess whether the effect estimates change if we use occasional users as the reference group. Seventh, we repeated the analyses by applying the 3-month preconception alcohol consumption in the miscarriage analyses, to harmonize categories and timeframes across sexes. Missing data on covariates ranged from 0.1% to 41.5% for the partner’s BMI. Missing values were imputed using multiple imputations by chained equations (R package mice, 3.19.0) to reduce bias. Multiple imputation reliably handles missing data up to 50% under the Missing at Random assumption [32,33]. All models used multiple imputed datasets with Rubin’s rules to pool estimates. Analyses were performed in R 4.2.3 (R Foundation for Statistical Computing, Vienna, Austria); *p* values from two-sided tests, with significance at *p* < 0.05.

## 3. Results

### 3.1. Participant Characteristics

Table 1 shows characteristics for time-to-pregnancy and miscarriage populations. Women who consumed, on average, no alcohol during the 12 months of preconception were more likely to be non-Dutch, lower educated, less likely to smoke or use illicit drugs, more likely to experience pregnancy loss, and had a longer time to pregnancy (Table S1). Supplementary Tables S2 and S3 show that women not included in the analyses consume alcohol, binge drink, and use illicit drugs more frequently during early pregnancy and are more often multiparous than those included in the analyses.

### 3.2. Fecundability and Infertility

**Figure 1:**
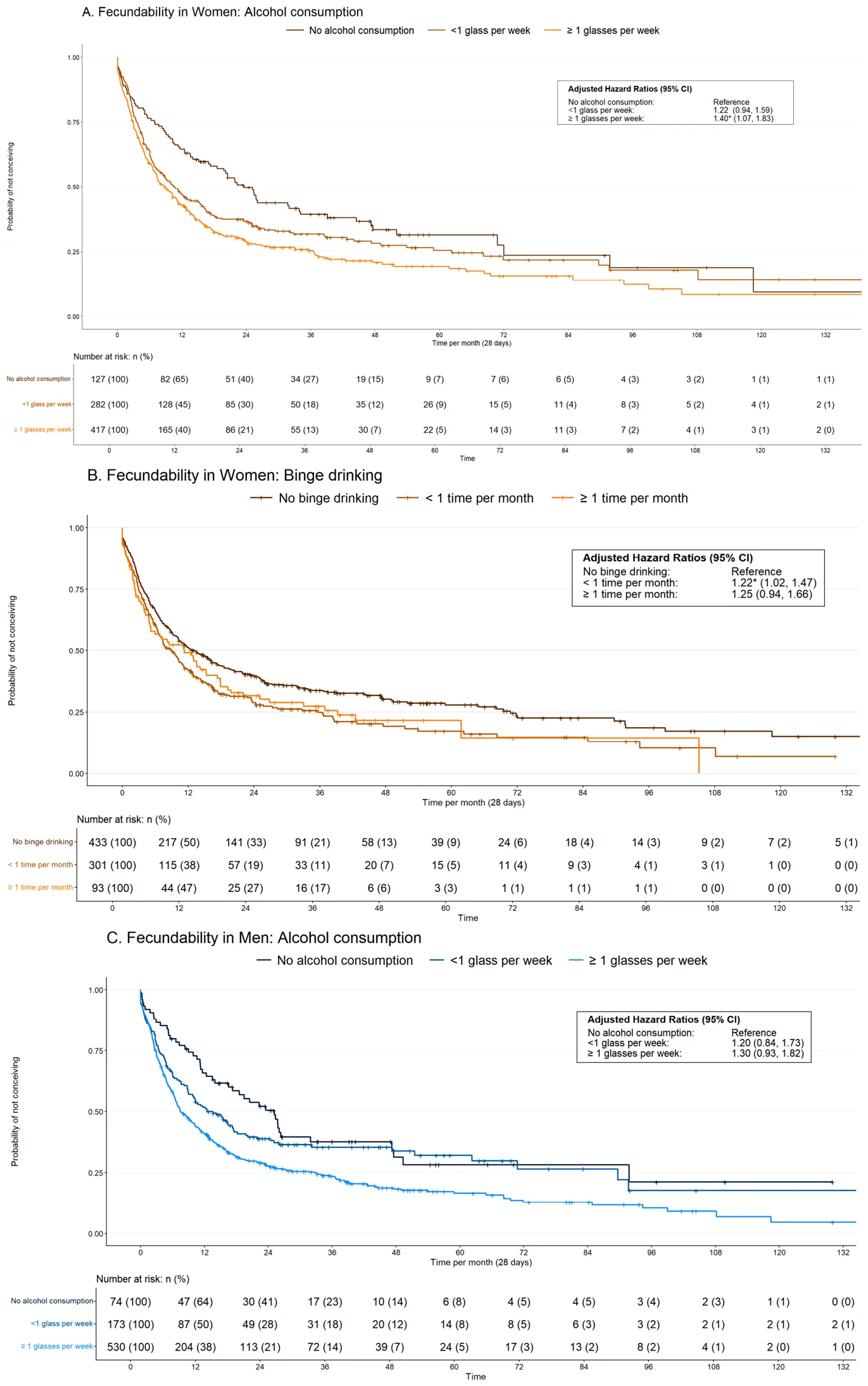

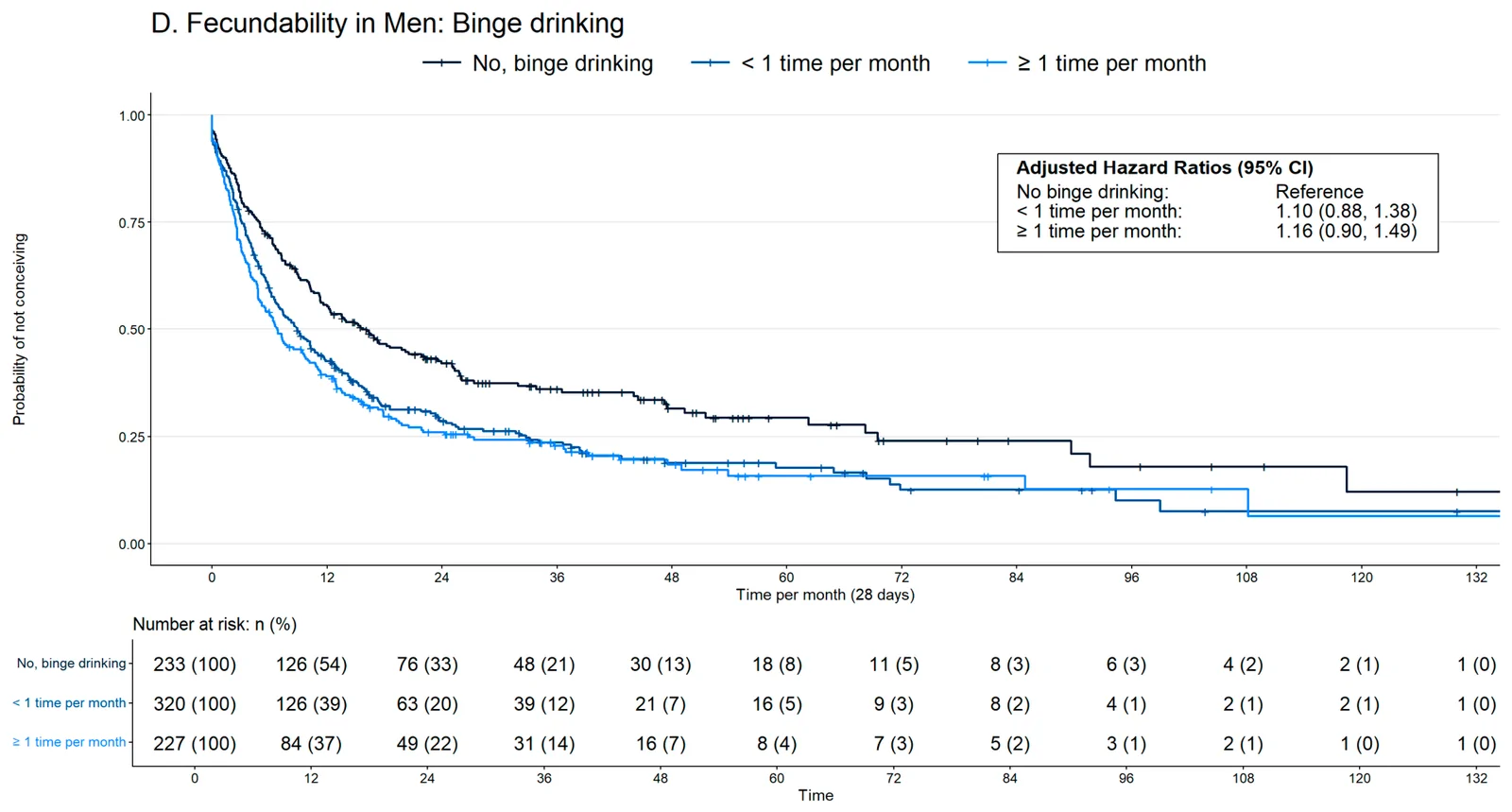
Preconceptional alcohol consumption and binge drinking with fecundability ratio. Fecundability ratios (FRs) with 95% CIs of alcohol consumption and binge drinking categories associated with fecundability. FRs were derived from the HRs of the Cox proportional hazards regression model. The FR was calculated as follows: HR = hazard rate [H(t)] of the different categories/[H(t) reference category]. * *p* value < 0.05. The main analyses are presented in Table S5. Survival curves were derived from the unadjusted models.

**Figure 2:**
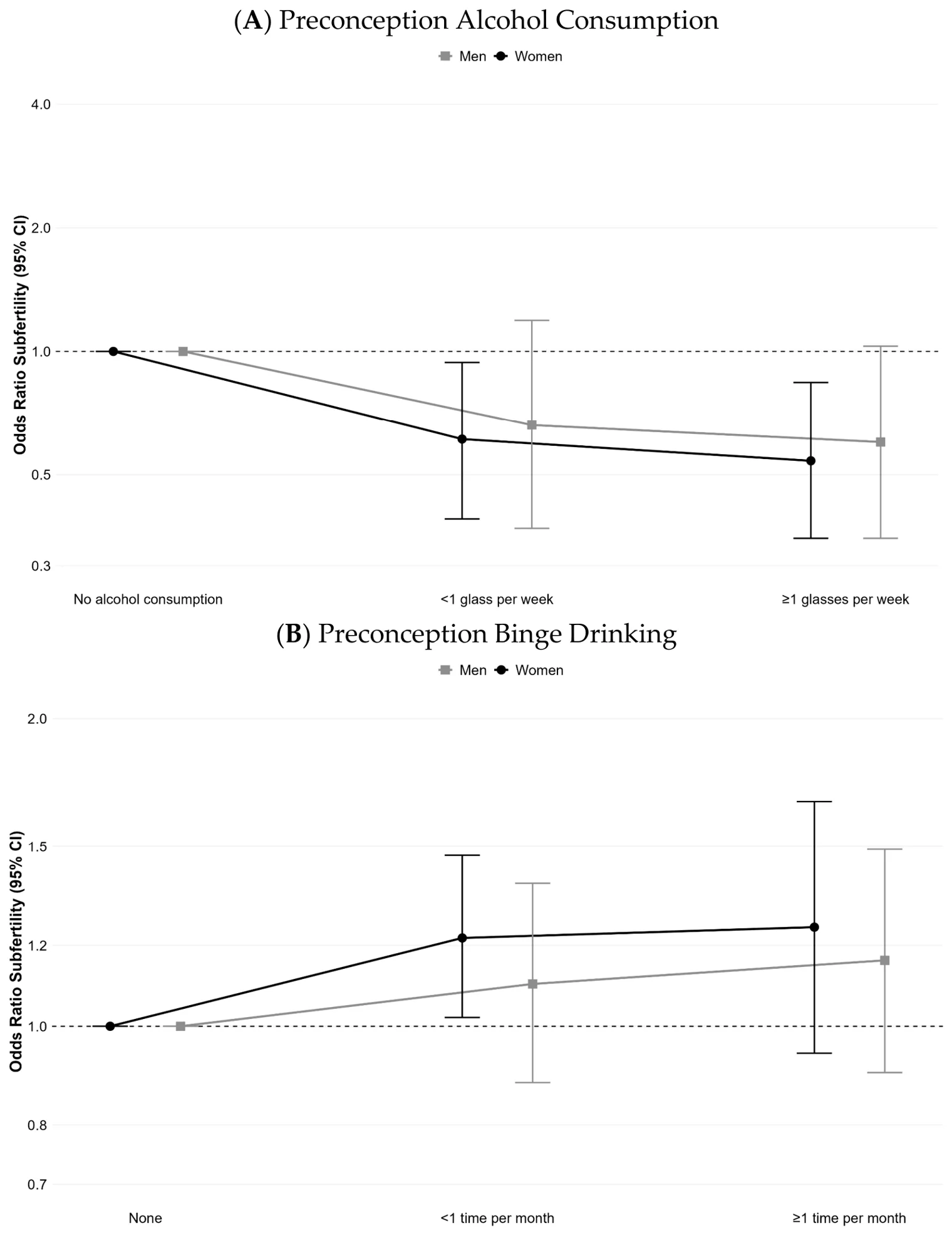
Preconceptional alcohol consumption and binge drinking and odds of infertility. Odds ratios (OR) (with 95% confidence interval) of infertility associated with preconceptional alcohol consumption and binge drinking in categories. ORs were derived from the exponentiated coefficients of the logistic regression models. An OR > 1 indicates increased odds of infertility as compared to the reference category, ‘No alcohol consumption’ or ‘None’. The main analyses are presented in Table 2.

Figure 1 shows that, as compared to women without preconceptional alcohol consumption, those who consumed ≥1 glass per week had increased fecundability (FR 1.40, 95% confidence interval (CI) 1.07, 1.83). As compared to women without preconceptional binge drinking, those with preconceptional binge drinking <1 time per month had increased fecundability (FR 1.22, 95% CI 1.02, 1.47). Both associations attenuated after adjusting for confounders (see Table S4). Figure 2 shows that, as compared to no alcohol consumption, preconceptional alcohol consumption of <1 glass per week and ≥1 glass per week among women reduced the odds of infertility (odds ratio (OR) 0.61, 95% CI 0.39, 0.94 and OR 0.54, 95% CI 0.35, 0.84, respectively). These effect sizes attenuated after full adjustment (see Table S9 for the basic models). Preconceptional binge drinking was associated with infertility in the basic model but attenuated after adjustment (Table 2 and Table S10). Among men, preconceptional alcohol consumption and binge drinking were not associated with fecundability and infertility in the adjusted models (Table 2; Table S5 presents the adjusted Cox regression model). An association was observed only in the basic model (see Table S4 for Cox regression and Table S10 for Logistic regression). Sensitivity analyses that excluded women who underwent assisted reproductive technology, excluded the top 5% of time to pregnancy, restricted analyses to only participants’ first episode, adjusted for partners’ alcohol consumption, or used alcohol consumption during the 3 months preconception did not materially change the effect estimates (Tables S6–S8 and S11–S15). Also, when we used occasional consumers (<1 glass per week and <1 binge-drinking episode per month) as the reference group, we observed that the observed associations remained consistent with results from the main analyses (Tables S9 and S16).

### 3.3. Miscarriage

**Figure 3:**
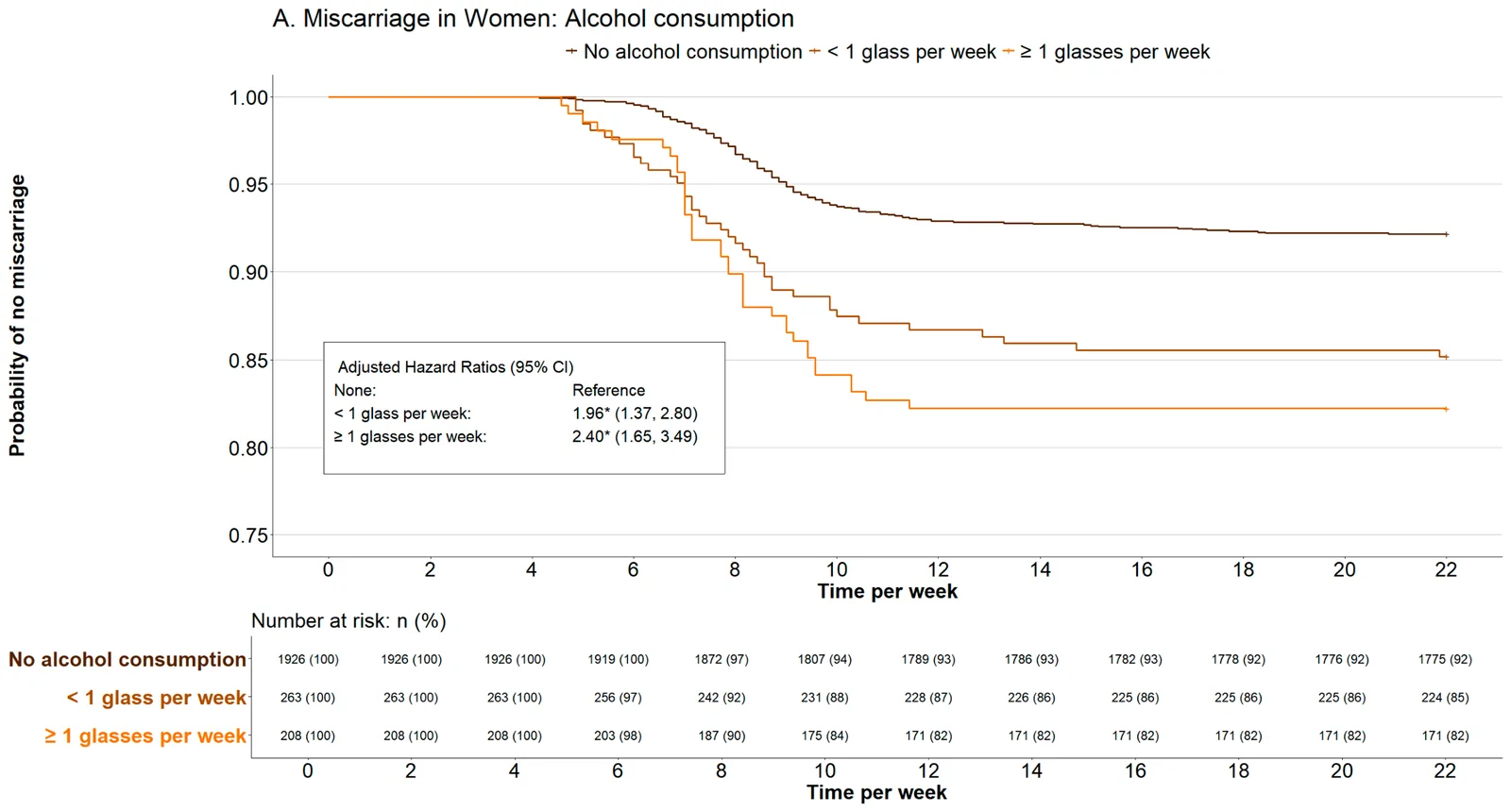

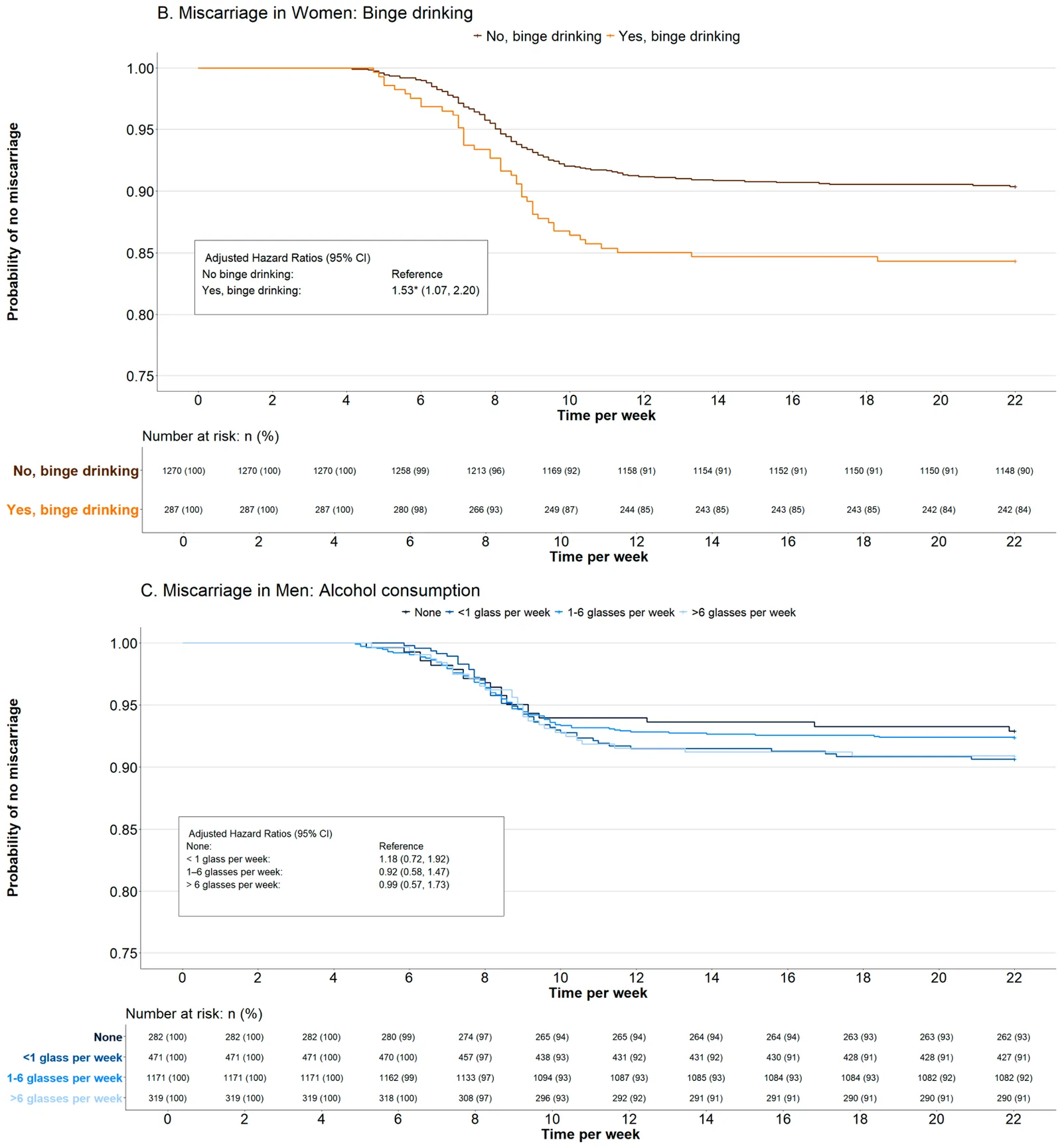

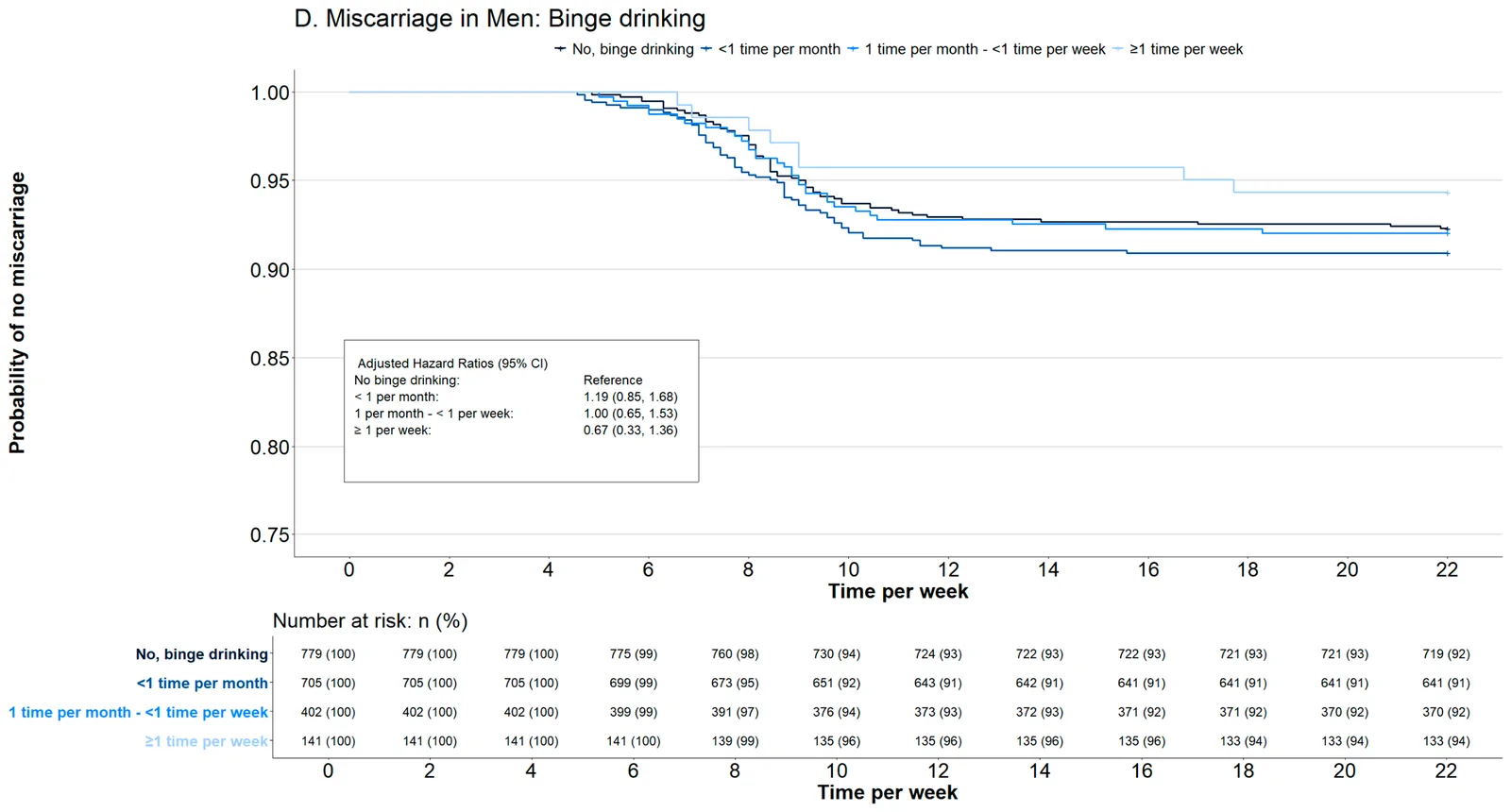
Early-pregnancy alcohol consumption, binge drinking, and miscarriage hazards. Hazard ratios (HRs) with 95% CIs of early-pregnancy alcohol consumption and binge drinking categories associated with miscarriage. The HR was calculated as follows: HR = hazard rate [H(t)] of the different categories/[H(t) reference category]. * *p* value < 0.05. The main analyses are presented in Table S14. Survival curves were derived from the unadjusted models.

**Figure 4:**
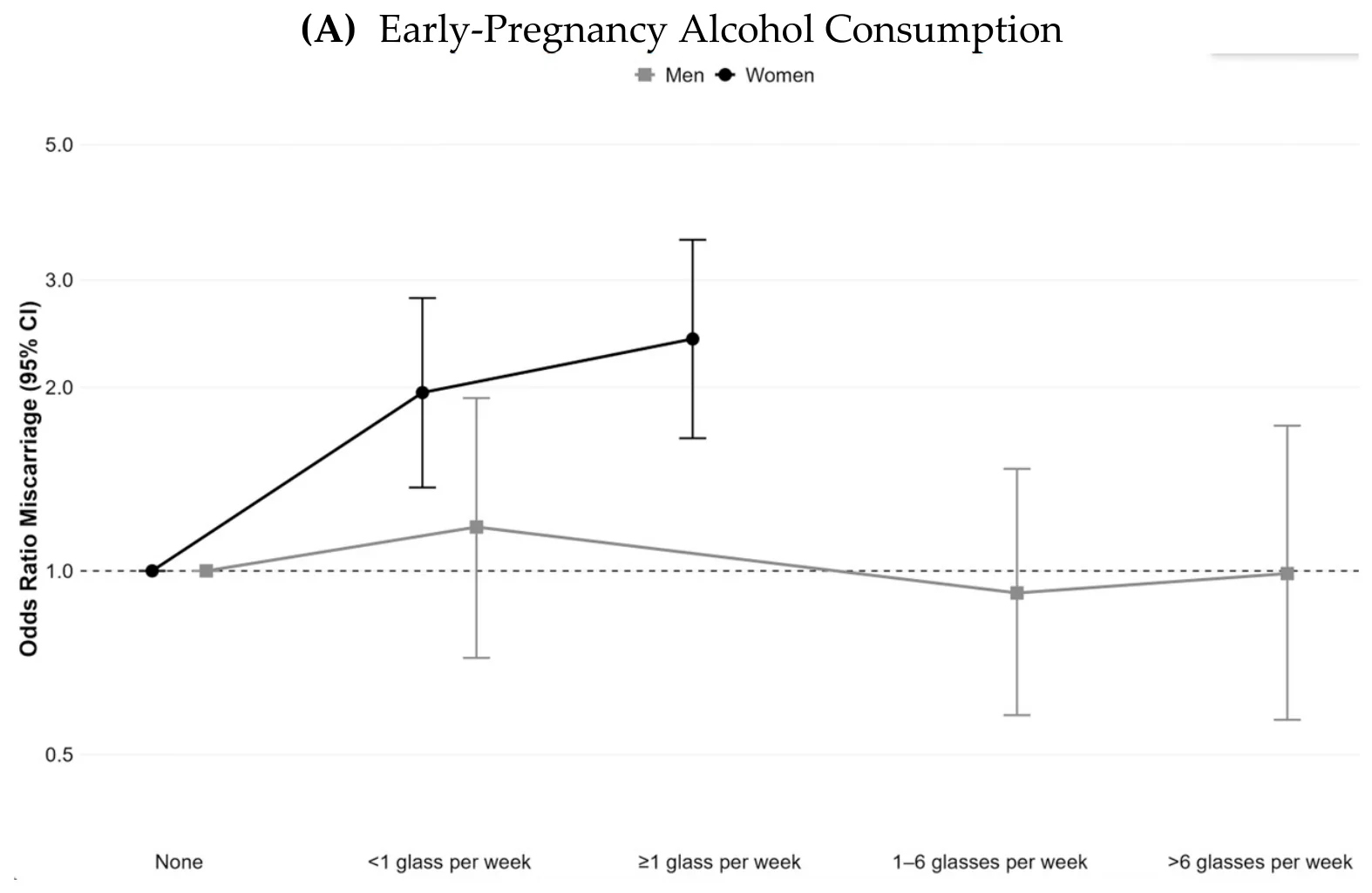

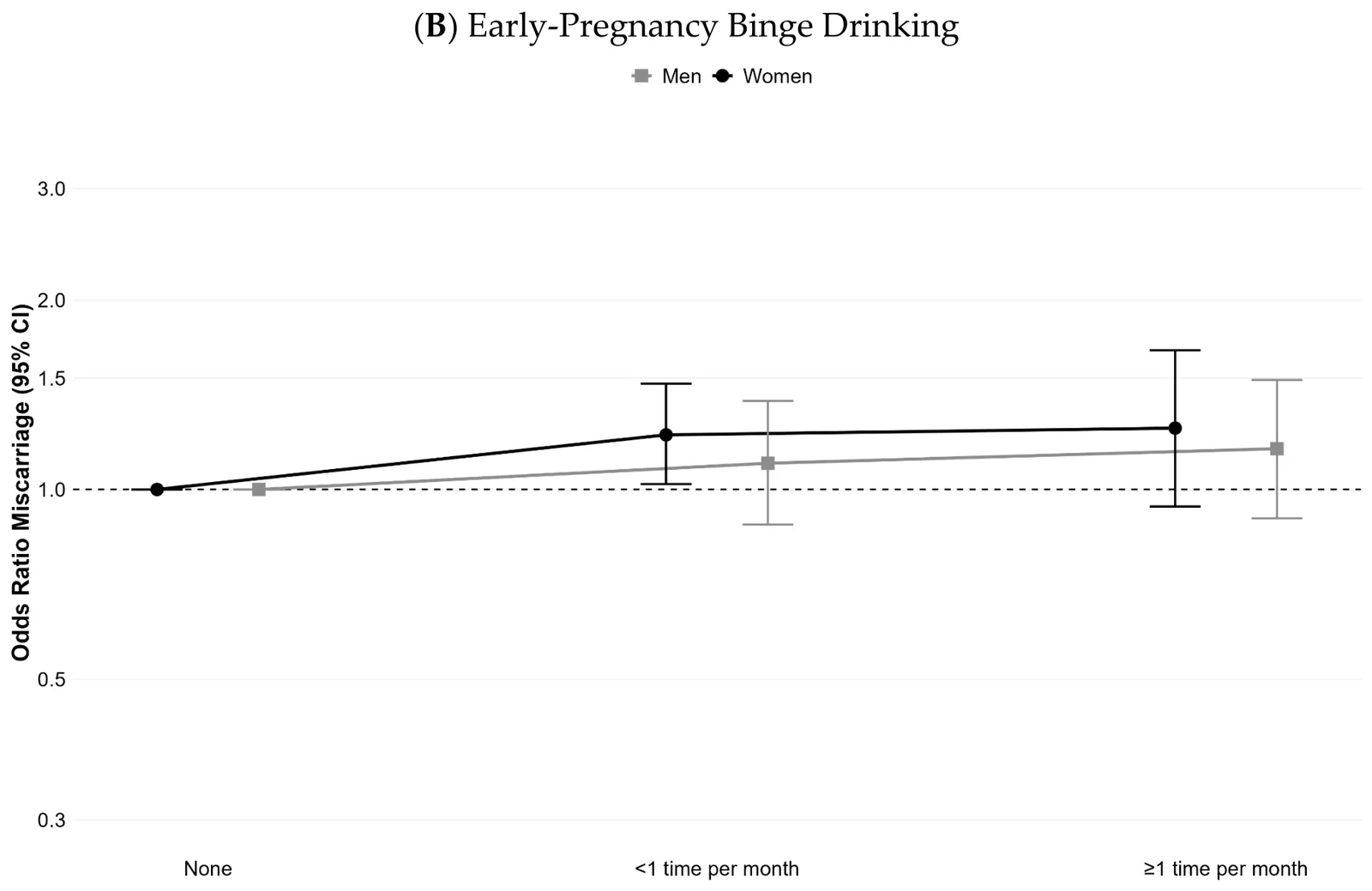
Early-pregnancy alcohol consumption, binge drinking, and miscarriage odds. Odds ratios (OR) (with 95% confidence intervals) of miscarriage associated with early-pregnancy alcohol consumption and binge drinking in categories. ORs were derived from the exponentiated coefficients of the logistic regression models. An OR > 1 indicates increased odds of miscarriage as compared to the reference category, ‘None’. The main analyses are presented in Table S19.

Figure 3 shows that, as compared to women without alcohol consumption in early pregnancy, those who consumed <1 glass per week and ≥1 glass per week had increased risks of miscarriage (Hazard ratio (HR) 1.96, 95% CI 1.37, 2.80, and HR 2.40, CI 1.65, 3.49, respectively). As compared to no binge drinking in early pregnancy among women, binge drinking increased the risk of miscarriage (HR 1.53, 95% CI 1.07, 2.20). In line with these findings, Figure 4 and Table 3 show that, as compared to no alcohol consumption in early pregnancy, drinking <1 glass per week or ≥1 glass per week increased the odds of miscarriage (adjusted OR 1.99, 95% CI 1.36, 2.92, and OR 2.37, 95% CI 1.58, 3.57, respectively). As compared to no binge drinking in early pregnancy among women, binge drinking increased the odds of miscarriage (adjusted OR 1.52, 95% CI 1.04, 2.24). Among men, early-pregnancy alcohol consumption and binge drinking were not associated with miscarriage in the adjusted and basic Cox regression and logistic regression models (Table 3, Tables S17, S18 and S24). Sensitivity analyses excluding women who underwent assisted reproductive technology, restricting analyses to only participants’ first episode, or adjusting for partners’ alcohol consumption did not materially change the effect estimates (Tables S19–S22 and S25–S28). In contrast to our main findings, sensitivity analyses that limited exposure to alcohol consumption in the three months before conception showed no association with miscarriage (Table S29).

## 4. Discussion

In this population-based prospective cohort study from the preconception period onwards, we observed that preconceptional low-to-moderate alcohol consumption among women was associated with higher fecundability and lower odds of infertility, whereas alcohol consumption during early pregnancy was consistently associated with an increased risk of miscarriage. Among men, alcohol consumption was not associated with infertility and miscarriage.

We observed that mild-to-moderate alcohol consumption in women was associated with greater fertility. This finding was unexpected and contrary to our hypothesis. These findings differ from earlier research on low-to-moderate alcohol consumption, which generally shows no effect at lower levels and only reduced fecundability at higher levels of consumption. A Danish cohort of 6120 women showed reduced fecundability only among those who consumed ≥14 glasses per week [34]. The Danish study adjusted for many confounders, yet it observed no consistent dose–response relationship. Additionally, a Spanish prospective cohort study of 7201 women found that alcohol consumption was not associated with reproductive outcomes after adjustment for socioeconomic and lifestyle factors, including education, BMI, smoking, physical activity, vitamin supplementation, and Mediterranean diet score, regardless of beverage type (beer, wine, or spirits) or consumption frequency (never or seldom, ≤1 per week, >1 to <5 per week, or ≥5 per week) [35]. Furthermore, a US-based cohort study of 413 women using daily diary data observed that moderate and heavy alcohol consumption (>3 glasses per week), particularly during the luteal and ovulatory phases, was associated with reduced fecundability compared with abstainers [36]. In contrast with these findings in women, we did not observe any association of alcohol consumption in men with fertility in our study.

Regarding women in our study, the observed association of preconceptional alcohol consumption with increased fecundability is unlikely to reflect a biological effect but may reflect residual confounding. Moderate alcohol consumers tend to have higher socioeconomic status and more favorable lifestyle factors than abstainers, including higher education, higher income, and lower BMI [17]. These factors are associated with greater fecundability [28,37]. Since our cohort mainly included highly educated women, unmeasured health-promoting behaviors associated with higher socioeconomic status and increased fertility might have caused residual confounding. In addition, sick-quitter bias may have resulted in confounding. Abstainers often include individuals who stopped consuming alcohol due to underlying fertility issues, potentially leading to overestimated associations within the reference group [38]. Consistent with this bias, our stratified baseline characteristics for alcohol consumption showed that women who reported no alcohol consumption were more likely to experience pregnancy loss and had a longer time to pregnancy as compared to women who consumed alcohol (Table S1). This might indicate differences in health between abstainers and alcohol consumers at baseline, rather than a direct effect of alcohol abstinence. To further assess this bias, we conducted a sensitivity analysis by repeating the primary models using occasional alcohol consumers (<1 glass/week and <1 time/month binge drinking) as the reference group. The associations observed remained largely consistent, which suggests that the characteristics of the reference group do not fully account for the observations of greater fertility among alcohol consumers.

In our study, early-pregnancy alcohol consumption among women, including low-to-moderate consumption and binge drinking, was associated with an increased risk of miscarriage. These findings support earlier research, which indicates that alcohol consumption during pregnancy, even in small amounts, increases the risk of miscarriage. Moreover, a large systematic review and meta-analysis of 24 studies, involving over 230,000 pregnancies, observed that alcohol consumption during pregnancy was associated with an increased risk of miscarriage [10]. Several studies included in the meta-analysis observed increased risk even with low-to-moderate alcohol consumption, while higher intake and binge drinking further increased the risk of miscarriage, suggesting a dose–response effect [10]. In contrast, another meta-analysis examined a subset of 5 studies from the 24 included in the earlier-mentioned review, including only studies that controlled for confounders and at least adjusted for maternal age [39]. This later meta-analysis also distinguished between first- and second-trimester miscarriage and did not observe a dose–response association.

Additionally, a US-based prospective cohort study including over 5000 women observed that each additional week of alcohol exposure during weeks 5 to 10 of gestation was associated with a higher risk of miscarriage [40]. Importantly, increased risks were observed even among women reporting low levels of alcohol consumption, indicating no safe level of alcohol consumption during pregnancy [40]. In contrast, an online US prospective cohort study of over 9000 women observed no association between preconception alcohol consumption and miscarriage [41]. Our research differs in that it focuses on the exposure window during early pregnancy and preconception. Additionally, we analyzed spontaneous miscarriages up to 22 weeks of gestation, considering ectopic pregnancies and terminations as censoring events because of their different biological mechanisms. Interestingly, both cohorts showed that abstainers generally had a less healthy baseline profile. We did not observe any associations of alcohol consumption in men with miscarriage in our study.

Biologically, preconception alcohol consumption, particularly at higher levels of consumption, may disrupt the hypothalamic–pituitary–gonadal axis [13]. In women, this affects the secretion of gonadotropins, particularly FSH and LH, and subsequently dysregulates the production of estradiol and progesterone [13]. These hormones are critical for follicular development and endometrial receptivity [13]. Endometrial receptivity is a key determinant of implantation; inadequate receptivity can prevent the blastocyst from implanting in the endometrium [14]. Similarly, in men, paternal alcohol consumption, particularly chronic consumption, may disrupt the hypothalamic–pituitary–gonadal axis [13]. This alters gonadotropin secretion, specifically FSH and LH, thereby dysregulating Leydig cells, which produce testosterone locally in the testes, leading to reduced intratesticular testosterone production [13]. These hormones are critical for testicular development and the maintenance of spermatogenesis [13]. Its alcohol-induced impairment can significantly reduce sperm concentration and alter sperm morphology [13,15]. Overall, this suggests that alcohol is more likely to decrease fecundability rather than improve it, supporting the view that conflicting findings in the literature are probably caused by residual confounding rather than a causal relationship. After successful implantation, the biological mechanism of alcohol shifts to direct toxicity of the embryo in utero, as it crosses the placenta by passive diffusion [42]. Alcohol induces oxidative stress and apoptosis, a process of programmed cell death, in embryonic cells, which may impair early development of the embryo [42]. The embryo during the first trimester is particularly vulnerable to the teratogenic effects of alcohol because organogenesis occurs between days 18 and 60 of gestation [42]. These mechanisms support the observed association between alcohol exposure during early pregnancy and increased miscarriage risk.

Strengths include a prospective cohort design with a sample size followed from preconception and early pregnancy onwards. This study assesses the independent as well as combined associations of maternal and paternal alcohol consumption during preconception and early pregnancy with fecundability, infertility, and miscarriage. This study has several limitations. First, non-response analyses showed that the included and excluded populations differed, potentially limiting the generalizability of the results to other populations. Excluded participants consumed alcohol, binge drank and used illicit drugs more frequently during early pregnancy and were more often multiparous than those included in the analyses. Second, the potential influence of the sick quitter bias must be considered, given that abstainers in our cohort systematically differ from those consuming alcohol. Abstainers had a higher baseline prevalence of prior miscarriage and a longer time to pregnancy. Using this group in our analyses may have underestimated the effect of preconceptional alcohol consumption, falsely suggesting it protects against infertility and increases fecundability. Third, alcohol consumption was self- or partner-reported and assessed via questionnaires, as commonly done in large cohort studies. Recall bias and social desirability bias may have caused misclassification, especially during early pregnancy [43]. Furthermore, early miscarriages could have been misclassified as non-conceptions, especially if the miscarriage occurred before clinical pregnancy recognition. Miscarriages are frequently undiagnosed as they are mistaken for heavy and late menstruation [44]. Potentially, if alcohol consumption increases the risk of unrecognized very early pregnancy loss, it would prolong the time to pregnancy and decrease the observed risk of miscarriage, leading to an overestimation of the association of alcohol with fecundability and infertility and an underestimation of the association of alcohol with miscarriage. Fourth, the questionnaires were collected before the miscarriage diagnosis, and no data collection was performed after the miscarriage diagnosis. However, the miscarriage might have already subclinically occurred before the diagnosis was made by ultrasound or clinical symptoms. Although this effect is expected to be minimal, it could potentially lead to reversed causality. Furthermore, the time windows for the alcohol assessments do not enable identification of time-specific effects during ovulation, implantation, or early pregnancy. Fifth, because higher alcohol consumption was rare in our study group, dividing it into more categories led to very small group sizes in the higher-intake categories, reducing statistical power. As a result, alcohol consumption was grouped broadly into none, less than one glass per week, and one or more glasses per week for the fertility analyses. This broad categorization combines different drinking habits and limits the ability to analyze detailed dose–response relationships. Finally, due to the observational nature of this cohort study, residual confounding cannot be completely excluded. Therefore, the statistical associations should be interpreted with caution, as they do not provide proof of a clinically protective effect of alcohol on reproductive outcomes.

## 5. Conclusions

The associations of low-to-moderate preconception alcohol consumption with lower odds of infertility might reflect healthier lifestyles, socioeconomic factors, and residual confounding by health-related abstinence from alcohol, all of which are associated with greater fertility. Therefore, preconception and early-pregnancy counseling should mainly focus on the risk of miscarriage and fetal health outcomes.

## Figures and Tables

**Table 1:** Characteristics of episodes of included participants.

	Episodes, No (%)			
	Time to Pregnancy		Miscarriage	
	Women (*n* = 945) ^a^	Men (*n* = 938) ^a^	Women (*n* = 2465) ^a^	Men (*n* = 2271) ^a^
Age at enrollment in years, median (IQR)	31.5 [29.5, 34.5]	33.4 [30.8, 36.5]	31.6 [29.2, 34.4]	33.5 [30.5, 36.8]
Missing	0 (0.0)	349 (36.9)	0 (0.0)	324 (13.1)
Ethnicity				
Dutch, *n* (%)	596 (63.2)	546 (67.7)	1582 (65.0)	1487 (64.2)
Non-Dutch, *n* (%) ^b^	347 (36.8)	261 (32.3)	853 (35.0)	830 (35.8)
Missing	2 (0.2)	138 (14.6)	30 (1.2)	148 (6.0)
Education level				
No, primary or secondary education, *n* (%)	206 (22.0)	286 (32.5)	655 (27.1)	839 (36.1)
Higher education, *n* (%)	729 (78.0)	594 (67.5)	1760 (72.9)	1486 (63.9)
Missing	10 (1.1)	65 (6.9)	50 (2.0)	140 (5.7)
Preconception alcohol consumption				
None, *n* (%)	142 (15.1)	80 (9.1)	-	-
<1 glass per week, *n* (%)	330 (35.1)	192 (21.8)	-	-
≥1 glass per week, *n* (%)	469 (49.8)	608 (69.1)	-	-
missing	4 (0.4)	65 (6.9)	-	-
Preconception binge drinking				
No binge drinking, *n* (%)	496 (52.7)	263 (29.8)	-	-
<1 time per month, *n* (%)	347 (36.8)	366 (41.4)	-	-
≥1 time per month *n* (%)	99 (10.5)	254 (28.8)	-	-
missing	3 (0.3)	62 (6.6)	-	-
Early-pregnancy alcohol consumption				
None, *n* (%)	-	-	1928 (80.3)	294 (12.8)
<1 glass per week, *n* (%)	-	-	265 (11.0)	483 (21.1)
≥1 glass per week, *n* (%)	-	-	208 (8.7)	-
1–6 glasses per week, *n* (%)	-	-	-	1192 (52.0)
>6 glasses per week, *n* (%)	-	-	-	324 (14.1)
missing	-	-	64 (2.6)	172 (7.0)
Early-pregnancy binge drinking				
No, binge drinking, *n* (%)	-	-	1271 (81.5)	798 (38.5)
Yes, binge drinking, *n* (%)	-	-	288 (18.5)	-
<1 time per month, *n* (%)	-	-	-	725 (35.0)
≥1 time per month, but <1 per week *n* (%)	-	-	-	408 (19.7)
≥1 time per week *n* (%)	-	-	-	143 (6.9)
missing	-	-	906 (36.8)	391 (15.9)
Preconception drug use				
No, *n* (%)	779 (82.9)	660 (73.8)	-	-
Yes, *n* (%)	161 (17.1)	234 (26.2)	-	-
Missing	5 (0.5)	51 (5.4)	-	-
Early-pregnancy drug use				
No, *n* (%)	-	-	2367 (96.7)	1804 (78.1)
Yes, *n* (%)	-	-	81 (3.3)	507 (21.9)
Missing	-	-	17 (0.7)	154 (6.2)
Preconception smoking			-	-
No, *n* (%)	565 (59.9)	456 (65.1)	-	-
Yes, *n* (%)	378 (40.1)	244 (34.9)	-	-
Missing	2 (0.2)	245 (25.9)	-	-
Early-pregnancy smoking			-	-
No smoking, *n* (%)	-	-	1248 (55.5)	1145 (54.2)
Quit smoking before pregnancy, *n* (%)	-	-	680 (30.2)	550 (26.0)
Smoked during pregnancy, *n* (%)	-	-	320 (14.2)	417 (19.7)
Missing	-	-	217 (8.8)	353 (14.3)
BMI, median (IQR)	23.8 [21.5, 26.9]	24.7 [22.8, 27.0]	23.3 [21.2, 26.3]	25.0 [23.0, 27.4]
Missing	0	392 (41.5)	6 (0.2)	420 (17.0)
Parity				
Nulliparous, *n* (%)	702 (74.3)	702 (42.8) ^d^	1555 (64.4)	1473 (64.4) ^d^
Multiparous, *n* (%)	242 (25.6)	167 (25.8) ^d^	861 (35.6)	814 (35.6) ^d^
Missing	1 (0.1)	297 (31.4) ^d^	49 (2.0)	178 (7.2) ^d^
Miscarriage in previous pregnancy				
No, *n* (%)	-	-	1781 (79.8)	1693 (80.3)
Yes, *n* (%)	-	-	451 (20.2)	416 (19.7)
Missing	-	-	233 (9.5)	356 (14.4)
Time to pregnancy, months, median (95% range) ^c^	5.8 [0.0, 68.2]	5.1 [0.0, 51.8] ^d^	-	-
0–12 months, *n* (%)	444 (47.0)	426 (65.6) ^d^	-	-
>12 months, *n* (%)	151 (16.0)	112 (17.3) ^d^	-	-
ART leading to pregnancy, *n* (%)	109 (11.5)	95 (14.6) ^d^	-	-
Not pregnant, *n* (%)	241 (25.5)	16 (2.5) ^d^	-	-
Missing	0	296 (31.3) ^d^	-	-
Occurrence of miscarriage				
Miscarriage, *n* (%)	-	-	232 (9.4)	187 (8.0) ^d^
No miscarriage, *n* (%)	-	-	2233 (90.6)	2142 (92.0) ^d^
Missing	-	-	0	136 (5.5) ^d^
Timing of miscarriage in weeks, median (IQR)	-	-	8.1 [7.0, 9.4]	8.3 [7.1, 9.4] ^d^
First trimester, *n* (%)	-	-	213 (91.8)	173 (92.5) ^d^
Second trimester, *n* (%)	-	-	16 (6.9)	12 (6.4) ^d^
Missing	-	-	3 (1.3)	2 (1.1) ^d^

Abbreviations: ART, assisted reproductive technology; BMI, body mass index (calculated as weight in kilograms divided by height in meters squared); NA, not applicable. Women were included in preconception and pregnancy between 2017 and 2021. Values are presented as median (IQR), median (95% range) or number of participants (valid %). ^a^ Study population of time to pregnancy and miscarriage consisting of 3160 unique women and 2272 unique men from Rotterdam. ^b^ Included: African; American, non-western; Asian, non-western; Chinese; Indonesian ethnicity, American, western; Asian, western; Cape Verdean; Dutch Antilles; European; German; Yugoslav; Moroccan; Oceanian; Polish; Surinamesel or Turkish. ^c^ Time to pregnancy in months was derived from pregnancy episodes with a natural conception. ^d^ Parity, miscarriage in previous pregnancy, time to pregnancy in months, occurrence of miscarriage, and timing of miscarriage in weeks in men were derived from their partner.

**Table 2:** Preconceptional alcohol consumption in women and men and odds of infertility.

		Women		Men
	*n*	Odds Ratio (95% CI)	*n*	Odds Ratio (95% CI)
Preconception alcohol use				
None	141	Reference	78	Reference
<1 glass per week	322	0.61 * (0.39, 0.94)	188	0.66 (0.37, 1.19)
≥1 glass per week	446	0.54 * (0.35, 0.84)	583	0.60 (0.35, 1.03)
Preconception binge drinking				
None	487	Reference	257	Reference
<1 per month	327	0.77 (0.56, 1.04)	346	0.78 (0.55, 1.12)
≥1 time per month	96	0.82 (0.51, 1.33)	247	0.71 (0.47, 1.06)

* *p* value < 0.05. Values represent the odds of infertility per categories of alcohol consumption or binge drinking (95% confidence interval (CI)) from logistic regression models, as compared to the reference category. Models were adjusted for body mass index, participant age, ethnicity, education level, smoking and drug use. Parity was included in the women’s model.

**Table 3:** Early-pregnancy alcohol consumption in women and men and odds of miscarriage.

		Women		Men
	*n*	Odds Ratio (95% CI)	*n*	Odds Ratio (95% CI)
Pregnancy alcohol use				
None	1926	Reference	290	Reference
<1 glass per week	263	1.99 * (1.36, 2.92)	474	1.16 (0.70, 1.93)
≥1 glass per week	208	2.37 * (1.58, 3.57)	-	-
1–6 glasses per week	-	-	1183	0.89 (0.55, 1.43)
>6 glasses per week	-	-	323	0.96 (0.54, 1.71)
Pregnancy binge drinking				
No, binge drinking	1270	Reference	798	Reference
Yes, binge drinking	287	1.52 * (1.04, 2.24)	-	-
<1 time per month	-	-	725	1.17 (0.82, 1.67)
≥1 time per month, but <1 per week	-	-	408	0.97 (0.62, 1.52)
≥1 time per week	-	-	143	0.64 (0.31, 1.34)

* *p* value < 0.05. Values represent the odds of miscarriage per categories of alcohol consumption or binge drinking (95% confidence interval (CI)) from logistic regression models, as compared to the reference category. Models were adjusted for body mass index, participant age, ethnicity, education level, smoking and drug use. Parity and history of miscarriage were included in the women’s model.

## Data Availability

Due to ethical, consent, and privacy restrictions, the data, analytical methods, and study materials cannot be made publicly available to other researchers for the purpose of reproducing the results or replicating the procedure.

## Supplementary Materials

The following supporting information can be downloaded at: https://www.mdpi.com/article/10.3390/nu18182953/s1, Method S1: Calculation of Fecundability Ratio on the Categorical Scale; Figure S1. Flowchart of Participants Included in the Analyses; Figure S2. Directed Acyclic Graph of Alcohol Consumption and Time to Pregnancy; Figure S3. Directed Acyclic Graph of Alcohol Consumption and Miscarriage; Table S1. Population Characteristics Presented per Level of Alcohol Consumption of Women; Table S2. Non-Response Analysis of Unique Participants Included and Excluded from the Study Populations; Table S3. Non-Response Analysis of Episodes Included and Excluded from the Study Population; Table S4. Associations of Preconceptional Alcohol Consumption in Women and Men with Fecundability Ratios, Basic Model; Table S5. Associations of Preconceptional Alcohol Consumption in Women and Men with Fecundability Ratios, Adjusted Model; Table S6. Associations of Preconceptional Alcohol Consumption in Women and Men with Fecundability Ratios, Adjusted Model Excluding the Top 5% of Time to Pregnancy; Table S7. Associations of Preconceptional Alcohol Consumption in Women and Men with Fecundability Ratios, Adjusted Model Including Only First Episodes; Table S8. Associations of Preconceptional Alcohol Consumption in Women and Men with Fecundability Ratios, Adjusted Model, Alcohol Consumption 3 Months Preconception; Table S9. Associations of Preconceptional Alcohol Consumption in Women and Men with Fecundability Ratios, Adjusted Model, Alcohol Consumption as the Reference Group; Table S10. Associations of Preconceptional Alcohol Consumption with the Odds of Infertility, Basic Model; Table S11. Associations of Preconceptional Alcohol Consumption in Women and Men with Odds of Infertility, Adjusted Model, Excluding Couples Undergoing Assisted Reproductive Technology; Table S12. Associations of Alcohol Consumption in Women and Men with Odds of Infertility, Adjusted Model Including Only First Episodes; Table S13. Associations of Alcohol Consumption in Women and Men with Odds of Infertility, Adjusted Model Excluding Top 5% of Time to Pregnancy; Table S14. Associations of Preconceptional Alcohol Consumption in Women and Men with Odds of Infertility, Adjusted Model Including Alcohol Consumption of Partner; Table S15. Associations of Preconceptional Alcohol Consumption in Women and Men with Odds of Infertility, Adjusted Model, 3 Months Preconception; Table S16. Associations of Preconceptional Alcohol Consumption with the Odds of Infertility, Adjusted Model, Alcohol Consumption as The Reference Group; Table S17. Associations Of Early-Pregnancy Alcohol Consumption in Women and Men with Hazard Ratios of Miscarriage, Basic Model; Table S18. Associations Of Early-Pregnancy Alcohol Consumption in Women and Men with Hazard Ratios of Miscarriage, Adjusted Model; Table S19. Associations Of Early-Pregnancy Alcohol Consumption in Women and Men with Hazard Ratios of Miscarriage, Adjusted Model Excluding Couples Undergoing Assisted Reproductive Technology; Table S20. Associations Of Early-Pregnancy Alcohol Consumption in Women and Men with Hazard Ratios of Miscarriage, Adjusted Model Including Only First Episodes; Table S21. Associations Of Early-Pregnancy Alcohol Consumption in Women and Men with Hazard Ratios of Miscarriage, Adjusted Model Including Alcohol Consumption of Partner; Table S22. Associations Of Early-Pregnancy Alcohol Consumption in Women and Men with Hazard Ratios of Miscarriage, Adjusted Model, 3 months preconception; Table S23. Associations Of Early-Pregnancy Alcohol Consumption in Women and Men with Hazard Ratios of Miscarriage, Adjusted Model, Alcohol Consumption as The Reference Group; Table S24. Associations Of Early-Pregnancy Alcohol Consumption in Women and Men with Odds of Miscarriage, Basic Model; Table S25. Associations Of Early-Pregnancy Alcohol Consumption in Women and Men with Odds of Miscarriage, Adjusted Model Excluding Couples Undergoing Assisted Reproductive Technology; Table S26. Associations Of Early-Pregnancy Alcohol Consumption in Women and Men with Odds of Miscarriage, Adjusted Model Including Only First Episodes; Table S27. Associations Of Early-Pregnancy Alcohol Consumption in Women and Men with Odds of Miscarriage, Adjusted Model Including Alcohol Consumption of Partner; Table S28. Associations Of Early-Pregnancy Alcohol Consumption in Women and Men with Odds of Miscarriage, adjusted model, 3 months preconception; Table S29. Associations Of Early-Pregnancy Alcohol Consumption in Women and Men with Odds of Miscarriage, Adjusted Model, Alcohol Consumption as The Reference Group; STROBE Statement—Checklist of Items That Should Be Included in Reports of Cohort Studies.

## Abbreviations

The following abbreviations are used in this manuscript:

BMI	Body mass index
CI	Confidence interval
DAG	Directed acyclic graph
FR	Fecundability ratio
HR	Hazard ratio
IVF	In vitro fertilization
IUI	Intrauterine insemination
OR	Odds ratio
