# Supplementary material for: Preconception and Early-Pregnancy Alcohol Consumption in Women and Men, Infertility and Risk for Miscarriage
Source: Nutrients. 2026 Sep 9;18(18):2953. doi: 10.3390/nu18182953 (PMC13610473; doi:10.3390/nu18182953)
Supplement: Supplementary file 1 [file nutrients-18-02953-s001.zip › nutrients-4475078-supplementary.pdf]

Supplementary Materials

Preconception and Early-pregnancy Alcohol Consumption in Women and Men, Infertility and Risk for Miscarriage

Short title: Periconceptional Alcohol Consumption and Fertility Outcomes

Morena Martinez Mayans[1,2], Celine H.X. Lin[1,2], Aline J. Boxem[1,2], Charlotte P. van Brienen[1,2], Annemarie G.M.G.J. Mulders[3], Romy Gaillard[1,2], Vincent W.V. Jaddoe[1,2]

1. The Generation R Study Group, Erasmus MC, University Medical Center, Rotterdam, the Netherlands.
2. Department of Pediatrics, Sophia's Children's Hospital, Erasmus MC, University Medical Center, Rotterdam, the Netherlands.
3. Department of Obstetrics and Gynecology, Sophia's Children's Hospital, Erasmus MC, University Medical Center, Rotterdam, the Netherlands.

Supplementary Content

Method S1: Calculation of Fecundability Ratio on the Categorical Scale 4

Figure S1. Flowchart of Participants Included in the Analyses 5

Figure S2. Directed Acyclic Graph of Alcohol Consumption and Time to Pregnancy. 6

Figure S3. Directed Acyclic Graph of Alcohol Consumption and Miscarriage. 7

Table S1. Population Characteristics Presented per Level of Alcohol Consumption of Women 8

Table S2. Non-Response Analysis of unique Participants Included and Excluded from the Study Populations 11

Table S3. Non-Response Analysis of Episodes Included and Excluded from the Study Population 14

Table S4. Associations Of Preconceptional Alcohol Consumption in Women and Men with Fecundability Ratios, Basic Model 17

Table S5. Associations Of Preconceptional Alcohol Consumption in Women and Men With Fecundability Ratios, Adjusted Model 18

Table S6. Associations of Preconceptional Alcohol Consumption in Women and Men with Fecundability Ratios, Adjusted Model Excluding the Top 5% of Time to Pregnancy 19

Table S7. Associations Of Preconceptional Alcohol Consumption in Women and Men with Fecundability Ratios, Adjusted Model Including Only First Episodes 20

Table S8. Associations Of Preconceptional Alcohol Consumption in Women and Men with Fecundability Ratios, Adjusted Model, Alcohol Consumption 3 Months Preconception. 21

Table S9. Associations Of Preconceptional Alcohol Consumption in Women and Men with Fecundability Ratios, Adjusted Model, Alcohol Consumption as The Reference Group 22

Table S10. Associations of Preconceptional Alcohol Consumption with the Odds of Infertility, Basic Model. 23

Table S11. Associations Of Preconceptional Alcohol Consumption in Women and Men with Odds of Infertility, Adjusted Model, Excluding Couples Undergoing Assisted Reproductive Technology 24

Table S12. Associations Of Alcohol Consumption in Women and Men with Odds of Infertility, Adjusted Model Including Only First Episodes 25

Table S13. Associations Of Alcohol Consumption in Women and Men with Odds of Infertility, Adjusted Model Excluding Top 5% of Time to Pregnancy 26

Table S14. Associations Of Preconceptional Alcohol Consumption in Women and Men With Odds of Infertility, Adjusted Model Including Alcohol Consumption Of Partner 27

Table S15. Associations of Preconceptional Alcohol Consumption in Women and Men with Odds of Infertility, Adjusted Model, 3 months preconception 28

Table S16. Associations of Preconceptional Alcohol Consumption with the Odds of Infertility, Adjusted Model, Alcohol Consumption as The Reference Group 29

Table S17. Associations Of Early-Pregnancy Alcohol Consumption in Women and Men with Hazard Ratios of Miscarriage, Basic Model 30

Table S18. Associations Of Early-Pregnancy Alcohol Consumption in Women and Men with Hazard Ratios of Miscarriage, Adjusted Model 31

Table S19. Associations Of Early-Pregnancy Alcohol Consumption in Women and Men with Hazard Ratios of Miscarriage, Adjusted Model Excluding Couples Undergoing Assisted Reproductive Technology 32

Table S20. Associations Of Early-Pregnancy Alcohol Consumption in Women and Men with Hazard Ratios of Miscarriage, Adjusted Model Including Only First Episodes 33

Table S21. Associations Of Early-Pregnancy Alcohol Consumption in Women and Men with Hazard Ratios of Miscarriage, Adjusted Model Including Alcohol Consumption of Partner 34

Table S22. Associations Of Early-Pregnancy Alcohol Consumption in Women and Men with Hazard Ratios of Miscarriage, Adjusted Model, 3 months preconception 35

Table S23. Associations Of Early-Pregnancy Alcohol Consumption in Women and Men with Hazard Ratios of Miscarriage, Adjusted Model, Alcohol Consumption as The Reference Group 36

Table S24. Associations Of Early-Pregnancy Alcohol Consumption in Women and Men with Odds of Miscarriage, Basic Model 37

Table S25. Associations Of Early-Pregnancy Alcohol Consumption in Women and Men with Odds of Miscarriage, Adjusted Model Excluding Couples Undergoing Assisted Reproductive Technology 38

Table S26. Associations Of Early-Pregnancy Alcohol Consumption in Women and Men with Odds of Miscarriage, Adjusted Model Including Only First Episodes 39

Table S27. Associations Of Early-Pregnancy Alcohol Consumption in Women and Men with Odds of Miscarriage, Adjusted Model Including Alcohol Consumption of Partner 40

Table S28. Associations Of Early-Pregnancy Alcohol Consumption in Women and Men with Odds of Miscarriage, adjusted model, 3 months preconception 41

Table S29. Associations Of Early-Pregnancy Alcohol Consumption in Women and Men with Odds of Miscarriage, Adjusted Model, Alcohol Consumption as The Reference Group 42

STROBE Statement—checklist of items that should be included in reports of observational studies 43

Method S1: Calculation of Fecundability Ratio on the Categorical Scale

The Hazard Ratio (HR) was calculated by dividing the hazard rate of conceiving within one month of the different categories of alcohol consumption by the hazard rate of the reference category of alcohol consumption level in blood:

HR = (H(t) ± alcohol consumption category) / (H(t) alcohol consumption reference category).

A HR < 1 indicates a lower fecundability as compared to the reference category of alcohol consumption.

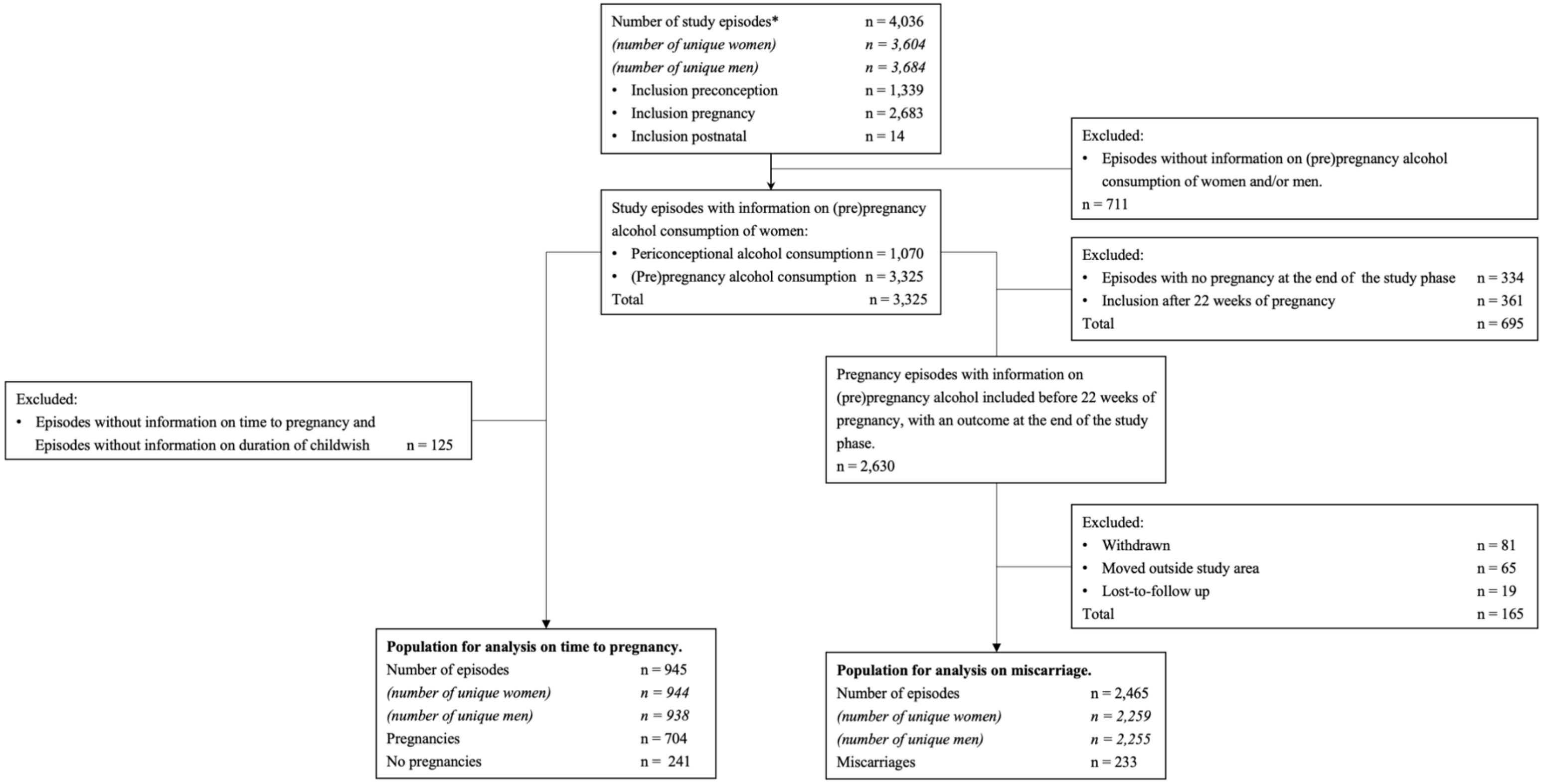

**Multiple episodes per participant were possible, consisting of the preconception phase, when applicable, and/or the pregnancy phase.*

Figure S1. Flowchart of Participants Included in the Analyses

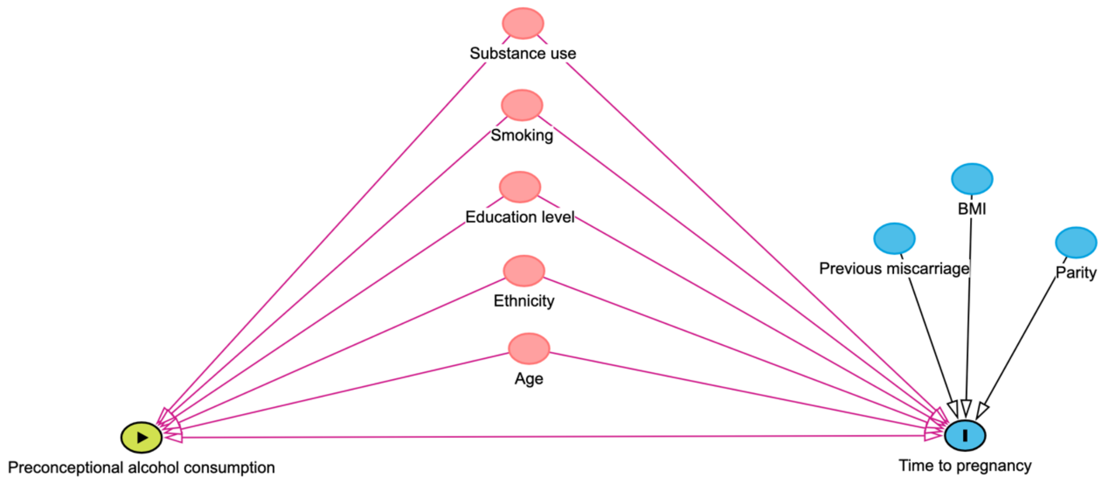

Figure S2. Directed Acyclic Graph of Alcohol Consumption and Time to Pregnancy.

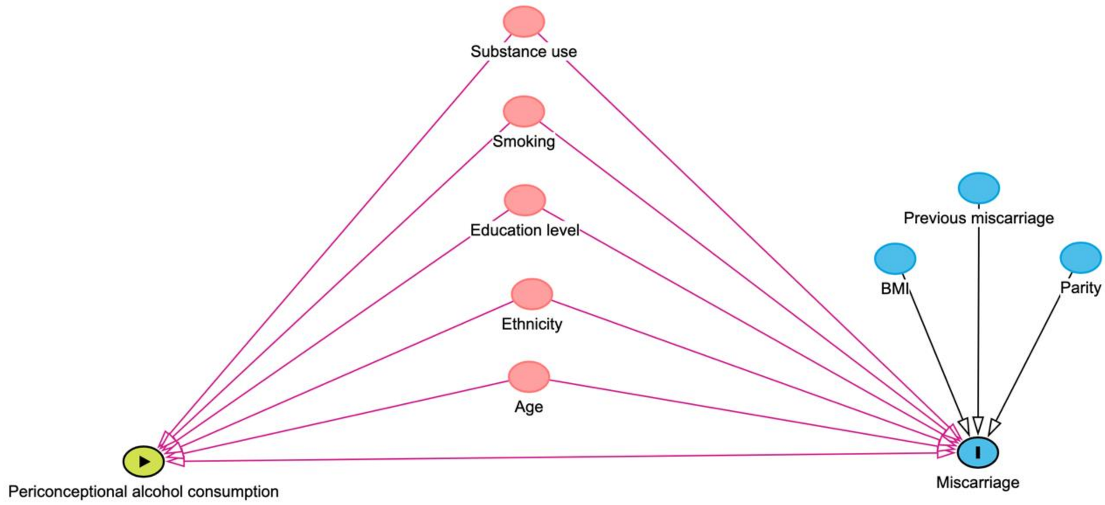

Figure S3. Directed Acyclic Graph of Alcohol Consumption and Miscarriage.

Table S1. Population Characteristics Presented per Level of Alcohol Consumption of Women

	Women			
	Episode, No %			
	No alcohol n=142	<1 glass per week n=330	≥ 1 glass per week n=469	p-value
Age at enrolment in years, median (IQR)	31.3 [29.0, 35.7]	31.8 [29.3, 35.2]	31.4 [29.8, 33.6]	0.910
Missing	0 (0.0)	0 (0.0)	0 (0.0)	
Ethnicity [A]				
Dutch, n (%)	56 (39.4)	188 (57.0)	350 (74.8)	<0.001
Non-Dutch, n (%)[c]	86 (60.6)	142 (43.0)	118 (25.2)	
Missing	0 (0.0)	0 (0.0)	1 (0.2)	
Education level				
No, primary or secondary education finished, n (%)	55 (39.6)	87 (26.8)	61 (13.1)	<0.001
Higher education finished, n (%)	84 (60.4)	238 (73.2)	406 (86.9)	
Missing	3 (2.1)	5 (1.5)	2 (0.4)	
Preconception binge drinking				
No binge drinking, n (%)	142 (100.0)	225 (68.4)	125 (26.8)	<0.001
< 1 time per month, n (%)	0 (0.0)	102 (31.0)	245 (52.5)	
≥ 1 time per month, n (%)	0 (0.0)	2 (0.6)	97 (20.8)	
missing	0 (0.0)	1 (0.3)	2 (0.4)	
Preconception drug use				
No, n (%)	135 (95.1)	285 (86.9)	357 (76.1)	<0.001
Yes, n (%)	7 (4.9)	43 (13.1)	112 (23.9)	
Missing	0 (0.0)	2 (0.6)	0 (0.0)	
Preconception smoking				
No, n (%)	105 (73.9)	205 (62.1)	253 (54.2)	<0.001
Yes, n (%)	37 (26.1)	125 (37.9)	214 (45.8)	

Missing	0 (0.0)	0 (0.0)	2 (0.4)	
BMI, median (IQR)	25.5 [23.1, 27.2]	25.0 [23.4, 27.3]	24.5 [22.5, 26.8]	0.021
Missing	0 (0.0)	0 (0.0)	0 (0.0)	
Parity				
Nulliparous, n (%)	90 (63.4)	226 (68.7)	383 (81.7)	<0.001
Multiparous, n (%)	52 (36.6)	103 (31.3)	86 (18.3)	
Missing	0 (0.0)	1 (0.3)	0 (0.0)	
Miscarriage in previous pregnancy				
No, n (%)	102 (71.8)	248 (75.2)	389 (82.9)	0.003
Yes, n (%)	40 (28.2)	82 (24.8)	80 (17.1)	
Missing	0 (0.0)	0 (0.0)		
Time to pregnancy in months, median (95% range) [B]	10.2 [0.0, 72]	5.6 [0.0, 62.5]	5.1 [0.0, 62.3]	0.007
0-12 months, n (%)	47 (33.1)	153 (46.4)	243 (51.8)	0.001
> 12 months, n (%)	34 (23.9)	46 (13.9)	71 (15.1)	
ART leading to pregnancy, n (%)	14 (9.9)	46 (13.9)	48 (10.2)	
Not pregnant, n (%)	47 (33.1)	85 (25.8)	107 (22.8)	
Missing	0 (0.0)	0 (0.0)	0 (0.0)	
Occurrence of miscarriage				
No miscarriage, n (%)	68 (71.6)	205 (83.7)	307 (84.8)	0.009
Miscarriage, n (%)	27 (28.4)	40 (16.3)	55 (15.2)	
Missing				
Timing of miscarriage in weeks, median (IQR)	8.0 [6.6, 8.4]	7.4 [6.3, 8.9]	8.1 [7.0, 9.4]	0.396
First trimester, n (%)	26 (96.3)	38 (95.0)	51 (92.7)	0.516
Second trimester, n (%)	1 (3.7)	1 (2.5)	4 (7.3)	
Missing	0 (0.0)	1 (2.5)	0 (0.0)	

Abbreviations: ART, assisted reproductive technology; BMI, body mass index (calculated as weight in kilograms divided by height in meters squared); NA, not applicable. Women were included in preconception and pregnancy between 2017 and 2021. Values are presented as median (IQR), median (95% range) or number of participants (valid %).

[A] Included: African; American, non-western; Asian, non-western; Chinese; Indonesian ethnicity, American, western; Asian, western; Cape Verdean; Dutch Antilles; European; German; Yugoslav; Moroccan; Oceanian; Polish; Surinamese, or Turkish.

[B] Time to pregnancy in months was derived from pregnancy episodes with a natural conception.

Table S2. Non-Response Analysis of unique Participants Included and Excluded from the Study Populations

	Women			Men		
	Participants, n (%)			Participants, n (%)		
	Responders n=2889[a]	Non-Responders N=143[b]	p-value	Responders n=2734[a]	Non-Responders n=106[b]	p-value
Age at enrolment in years, median (IQR)	31.5 [29.1, 34.3]	30.2 [27.4, 32.9]	<0.001	33.3 [30.4, 36.7]	33.6 [26.4, 35.5]	0.104
Missing	0 (0.0)	0 (0.0)		654 (22.6)	114 (80.0)	
Ethnicity						
Dutch, n (%)	1750 (61.3)	63 (49.6)	0.011	1646 (61.9)	52 (51.0)	0.033
Non-Dutch, n (%)[c]	1103 (38.7)	64 (50.4)		1011 (38.1)	50 (49.0)	
Missing	36 (1.2)	16 (11.2)		232 (8.0)	41 (28.7)	
Education level						
No, primary or secondary education, n (%)	832 (29.4)	34 (27.2)	0.670	1056 (39.2)	43 (39.1)	1.000
Higher education, n (%)	1999 (70.6)	91 (72.8)		1638 (60.8)	67 (60.9)	
Missing	58 (2.0)	18 (12.6)		195 (6.7)	33 (23.1)	
Preconception alcohol consumption						
None, n (%)	135 (14.5)	11 (12.0)	0.740	74 (8.5)	7 (8.6)	0.502
< 1 glass per week, n (%)	326 (35.0)	35 (38.0)		189 (21.6)	22 (27.2)	
≥ 1 glass per week, n (%)	470 (50.5)	46 (50.0)		611 (69.9)	52 (64.2)	
missing	1958 (67.8)	51 (35.7)		2015 (69.7)	62 (43.4)	
Preconception binge drinking,						
No binge drinking, n (%)	485 (52.0)	40 (43.0)	0.212	257 (29.3)	24 (29.3)	0.792
< 1 time per month, n (%)	348 (37.3)	43 (46.2)		367 (41.8)	37 (45.1)	
≥ 1 time per month, n (%)	99 (10.6)	10 (10.8)		253 (28.8)	21 (25.6)	
missing	1957 (67.7)	50 (35.0)		2012 (69.6)	61 (42.7)	
Early-pregnancy alcohol consumption						
None, n (%)	2155 (75.8)	28 (24.1)	<0.001	376 (13.9)	14 (13.5)	0.166
< 1 glass per week, n (%)	362 (12.7)	41 (35.3)		585 (21.6)	28 (26.9)	
≥ 1 glass per week, n (%)	327 (11.5)	47 (40.5)				
1-6 glasses per week, n (%)	-	-		1371 (50.7)	55 (52.9)	

>6 glasses per week, n (%)	-	-		373 (13.8)	7 (6.7)	
missing	45 (1.6)	27 (18,9)		184 (6.4)	39 (27.3)	
Early pregnancy binge drinking						
No, binge drinking, n (%)	1455 (78.5)	77 (59.2)	<0.001	1046 (39.7)	34 (34.0)	0.659
Yes, binge drinking, n (%)	398 (21.5)	53 (40.8)		-	-	
< 1 time per month, n (%)	-	-		912 (34.6)	40 (40.0)	
≥ 1 time per month, but <1 per week n (%)	-	-		495 (18.8)	19 (19.0)	
≥1 time per week n (%)	-	-		180 (6.8)	7 (7.0)	
missing	1036 (35.)	13 (9.1)		256 (8.9)	43 (30.1)	
Preconception drug use[d]						
No, n (%)	771 (82.3)	68 (73.1)	0.042	654 (73.6)	49 (62.0)	0.038
Yes, n (%)	166 (17.7)	25 (26.9)		235 (26.4)	30 (38.0)	
Missing	1952 (67.6)	50 (35.0)		2000 (69.2)	64 (44.8)	
Early pregnancy drug use[d]						
No, n (%)	2739 (95.1)	111 (79.9)	<0.001	2081 (76.3)	65 (64.4)	0.009
Yes, n (%)	140 (4.9)	28 (20.1)		648 (23.7)	36 (35.6)	
Missing	10 (0.3)	4 (2.8)		160 (5.5)	42 (29.4)	
Preconception smoking						
No, n (%)	1581 (55.5)	66 (53.7)	0.761	1334 (55.5)	49 (83.1)	<0.001
Yes, n (%)	1269 (44.5)	57 (46.3)		1071 (44.5)	10 (16.9)	
Missing	39 (1.3)	20 (14.0)		484 (16.8)	84 58.7)	
Early pregnancy smoking						
No, n (%)	1581 (55.5)	66 (53.7)	0.274	1334 (55.5)	49 (83.1)	<0.001
Quit smoking before pregnancy, n (%)	881 (30.9)	45 (36.6)		581 (24.2)	3 (5.1)	
Smoked during pregnancy, n (%)	388 (13.6)	12 (9.8)		490 (20.4)	7 (11.9)	
Missing	39 (1.3)	20 (14)		484 (16.8)	84 (58.7)	
BMI, median (IQR)	23.5 [21.2, 26.5]	23.8 [21.7, 27.3]	0.189	24.9 [23.0, 27.4]	24.6 [22.4, 28.8]	0.752
Missing	23 (0.8)	21 (15.0)		745 (25.8)	121 (85.0)	
Parity						
Nulliparous, n (%)	1925 (67.9)	97 (82.9)	0.001	1654 (67.3)	20 (74.1)	0.588

Multiparous, n (%)	910 (32.1)	20 (17.1)		804 (32.7)	7 (25.9)	
Missing	54 (1.9)	26 (18.2)		431 (14.9)	116 (81.1)	
Miscarriage in previous pregnancy						
No, n (%)	2303 (80.8)	104 (88.1)	0.062	2006 (81.2)	25 (89.3)	0.396
Yes, n (%)	546 (19.2)	14 (11.9)		465 (18.8)	3 (10.7)	
Missing	40 (1.4)	25 (17.5)		418 (14.5)	115 (80.4)	
Time to pregnancy in months, median (95% range) [d]	3.4 (0.0, 66.6)	-	-	3.2 (0.0, 59.3)	-	-
0-12 months, n (%)	1819 (65.5)	0 (0.0)	-	1777 (73.8)	0 (0.0)	-
> 12 months, n (%)	459 (16.5)	0 (0.0)		395 (16.4)	0 (0.0)	
ART leading to pregnancy, n (%)	263 (9.5)	0 (0.0)		225 (9.3)	0 (0.0)	
Not pregnant, n (%)	235 (8.5)	91 (100.0)		12 (0.5)	7 (100.0)	
Missing	611 (21.1)	52 (36.4)		717 (24.8)	136 (95.1)	
Occurrence of miscarriage						
No miscarriage, n (%)	2446 (92.2)	52 (100.0)	0.066	2328 (93.4)	44 (100.0)	0.147
Miscarriage, n (%)	208 (7.8)	0 (0.0)		164 (6.6)	0 (0.0)	
Missing	235 (8.1)	91 (63.6)		397 (13.7)	99 (69.2)	
Timing of miscarriage in weeks, median (IQR)	8.1 [7.0, 9.5]	-	-	8.4 [7.1, 9.6]	-	-
First trimester, n (%)	193 (92.8)	-	-	153 (93.3)	-	-
Second trimester, n (%)	13 (6.2)	-		10 (6.1)	-	
Missing	2 (1.0)	-		1 (0.6)	-	

Abbreviations: ART, assisted reproductive technology; BMI, body mass index (calculated as weight in kilograms divided by height in meters squared); NA, not applicable. Women were included in preconception and pregnancy between 2017 and 2021. Values are presented as median (IQR), median (95% range) or number of participants (valid %).

[A] Study population of time to pregnancy and miscarriage consisting of 3,160 unique women and 2,272 unique men from Rotterdam.

[B] Non-responders consisted of 238 unique women and 39 unique men.

[C] Included: African; American, non-western; Asian, non-western; Chinese; Indonesian ethnicity, American, western; Asian, western; Cape Verdean; Dutch Antilles; European; German; Yugoslav; Moroccan; Oceanian; Polish; Surinamese, or Turkish.

[d] Time to pregnancy in months was derived from pregnancy episodes with a natural conception.

[e] Parity, miscarriage in previous pregnancy, time to pregnancy in months, occurrence of miscarriage, timing of miscarriage in weeks in men were derived from their partner.

Table S3. Non-Response Analysis of Episodes Included and Excluded from the Study Population

	Women			Men		
	Episodes, No. %			Episodes, No. %		
	Responders n=2935[a]	Non-Responders n=155[b]	p-value	Responders n=2929[a]	Non-Responders n=9155[b]	p-value
Age at enrolment in years, median (IQR)	31.6 [29.2, 34.5]	30.3 [27.4, 33.1]	<0.001	33.5 [30.5, 36.8]	32.7 [28.3, 35.5]	0.046
Missing	0 (0.0)	0 (0.0)		680 (21.5)	114 (73.5)	
Ethnicity						
Dutch, n (%)	1952 (62.3)	71 (51.4)	0.022	1828 (62.5)	56 (50.0)	0.025
Non-Dutch, n (%)[c]	1181 (37.7	67 (48.6)		1096 (37.5)	56 (50.0)	
Missing	37 (1.2)	17 (11.0)		246 (7.8)	43 (27.7)	
Education level						
No, primary or secondary education finished, n (%)	895 (28.8)	39 (28.7)	1.000	1130 (38.1)	47 (39.2)	0.895
Higher education finished, n (%)	2216 (71.2)	97 (71.3)		1833 (61.9)	73 (60.8)	
Missing	59 (1.9)	19 (12.3)		207 (6.5)	35 (22.6)	
Preconception alcohol consumption						
None, n (%)	146 (15.1)	11 (12.0)	0.685	81 (8.9)	7 (8.6)	0.501
< 1 glass per week, n (%)	340 (35.2)	35 (38.0)		196 (21.5)	22 (27.2)	
≥ 1 glass per week, n (%)	481 (49.7)	46 (50.0)		633 (69.6)	52 (64.2)	
missing	2203 (69.5)	63 (40.6)		2266 (68.2)	74 (47.7)	
Preconception binge drinking,						
No binge drinking, n (%)	508 (52.5)	40 (43.0)	0.179	270 (29.6)	24 (29.3)	0.789
< 1 time per month, n (%)	357 (36.9)	43 (46.2)		380 (41.7)	37 (45.1)	
≥ 1 time per month, n (%)	103 (10.6)	10 (10.8)		262 (28.7)	21 (25.6)	
missing	2202 (69.5)	62 (40.0)		2331 (70.1)	74 (47.7)	
Early-pregnancy alcohol consumption						
None, n (%)	2361 (76.4)	33 (26.8)	<0.001	400 (13.6)	15 (14.0)	0.182
< 1 glass per week, n (%)	386 (12.5)	42 (34.1)		639 (21.7)	28 (26.2)	
≥ 1 glass per week, n (%)	342 (11.1)	48 (39.0)		-	-	
1-6 glasses per week, n (%)	-	-		1504 (51.1)	57 (53.3)	

>6 glasses per week, n (%)	-	-		399 (13.6)	7 (6.5)	
missing	81 (2.6)	32 (20.6)		276 (8.3)	48 (31.0)	
Early pregnancy binge drinking						
No, binge drinking, n (%)	1611 (79.6)	84 (61.3)	<0.001	1073 (39.8)	34 (34.0)	0.648
Yes, binge drinking, n (%)	413 (20.4)	53 (38.7)		-	-	
< 1 time per month, n (%)	-	-		932 (34.6)	40 (40.0)	
≥ 1 time per month, but <1 per week n (%)	-	-		509 (18.9)	19 (19.0)	
≥1 time per week n (%)	-	-		181 (6.7)	7 (7.0)	
missing	1146 (36.2)	18 (11.6)		530 (15.9)	55 (35.5)	
Preconception drug use						
No, n (%)	805 (82.8)	67 (73.6)	0.041	683 (74.0)	49 (62.0)	0.030
Yes, n (%)	167 (17.2)	24 (26.4)		240 (26.0)	30 (38.0)	
Missing	2198 (69.3)	64 (41.3)		2247 (70.9)	76 (49.0)	
Early pregnancy drug use						
No, n (%)	3006 (95.5)	122 (81.9)	<0.001	2288 (77.2)	67 (64.4)	0.004
Yes, n (%)	141 (4.5)	27 (18.1)		675 (22.8)	37 (35.6)	
Missing	23 (0.7)	6 (3.9)		207 (6.5)	51 (32.9)	
Preconception smoking						
No, n (%)	1613 (55.0)	66 (52.4)	0.627	1469 (55.9)	49 (79.0)	<0.001
Yes, n (%)	1320 (45.0)	60 (47.6)		1158 (44.1)	13 (21.0)	
Missing						
Early-pregnancy smoking						
No, n (%)	1613 (55.0)	66 (52.4)	0.559	1469 (55.9)	49 (79.0)	0.001
Quit smoking before pregnancy, n (%)	919 (31.3)	45 (35.7)		642 (24.4)	5 (8.1)	
Smoked during pregnancy, n (%)	401 (13.7)	15 (11.9)		516 (19.6)	8 (12.9)	
Missing	237 (7.5)	29 (18.7)		543 (17.1)	93 (60.0)	
BMI, median (IQR)	23.5 [21.2, 26.5]	23.8 [21.6, 27.3]	0.223	25.0 [23.0, 27.4]	24.3 [22.3, 28.1]	0.337
Missing	27 (0.9)	25 (16.1)		794 (25.0)	124 (80.0)	
Parity						
Nulliparous, n (%)	2028 (65.1)	98 (76.6)	0.010	1748 (64.2)[e]	21 (55.3)	0.331

Multiparous, n (%)	1085 (34.9)	30 (23.4)		974 (35.8)[e]	17 (44.7)	
Missing	57 (1.8)	27 (17.4)		448 (14.1)[e]	117 (75.5)	
Miscarriage in previous pregnancy						
No, n (%)	2312 (79.3)	104 (88.1)	0.026	2015 (79.6)[e]	25 (89.3)	0.303
Yes, n (%)	604 (20.7)	14 (11.9)		516 (20.4)[e]	3 (10.7)	
Missing	254 (8.0)	37 (23.9)		639 (20.2)[e]	127 (81.9)	
Time to pregnancy in months, median (95% range) [d]	3.7 [1.1, 11.0]	-	-	3.5 [1.0, 10.1][e]	-	-
0-12 months, n (%)	1938 (64.4)	0 (0.0)		1894 (71.9)[e]	0 (0.0)	
> 12 months, n (%)	540 (18.0)	0 (0.0)		475 (18.0)[e]	0 (0.0)	
ART leading to pregnancy, n (%)	286 (9.5)	0 (0.0)		248 (9.4)[e]	0 (0.0)	
Not pregnant, n (%)	243 (8.1)	91 (100.0)		16 (0.6)[e]	7 (100.0)	
Missing	163 (5.1)	64 (41.3)		537 (16.9)[e]	148 (95.5)	
Occurrence of miscarriage						
No miscarriage, n (%)	2695 (92.1)	64 (100.0)	-	2568 (93.2)[e]	56 (100.0)	-
Miscarriage, n (%)	232 (7.9)	0 (0.0)		187 (6.8)[e]	0 (0.0)	
Missing	243 (7.7)	91 (58.7)		415 (13.1)[e]	99 (63.9)	
Timing of miscarriage in weeks, median (IQR)	8.1 [7.0, 9.4]	-	-	8.3 [7.1, 9.4][e]	-	-
First trimester, n (%)	213 (91.8)	0 (0.0)		173 (92.5)[e]	0 (0.0)	
Second trimester, n (%)	16 (6.9)	0 (0.0)		12 (6.4)[e]	0 (0.0)	
Missing	2938 (92.7)	155 (100.0)		2983 (94.1)[e]	155 (100.0)	

Abbreviations: ART, assisted reproductive technology; BMI, body mass index (calculated as weight in kilograms divided by height in meters squared); NA, not applicable. Women were included in preconception and pregnancy between 2017 and 2021. Values are presented as median (IQR), median (95% range) or number of participants (valid %).

[A] Study population of time to pregnancy and miscarriage consisting of 3,160 unique women and 2,272 unique men from Rotterdam.

[B] Non-responders consisted of 238 unique women and 39 unique men.

[C] Included: African; American, non-western; Asian, non-western; Chinese; Indonesian ethnicity, American, western; Asian, western; Cape Verdean; Dutch Antilles; European; German; Yugoslav; Moroccan; Oceanian; Polish; Surinamese, or Turkish.

[d] Time to pregnancy in months was derived from pregnancy episodes with a natural conception.

[e] Parity, miscarriage in previous pregnancy, time to pregnancy in months, occurrence of miscarriage, timing of miscarriage in weeks in men were derived from their partner.

Table S4. Associations Of Preconceptional Alcohol Consumption in Women and Men with Fecundability Ratios, Basic Model

		Women		Men
	n	Fecundability ratio (95% CI)	n	Fecundability ratio (95% CI)
**Preconception alcohol use**				
None	127	Reference	74	Reference
<1 glass per week	282	1.33* (1.03, 1.72)	173	1.26 (0.90, 1.78)
≥1 glass per week	417	1.62* (1.27, 2.07)	530	1.77* (1.30, 2.40)
**Preconception binge drinking**				
None	433	Reference	233	Refer*ence*
<1 per month	301	1.32* (1.11, 1.57)	320	1.42* (1.16, 1.74)
≥ 1 time per month	93	1.27 (0.98, 1.65)	227	1.55* (1.25, 1.92)

*P value <0.05. Values represent the fecundability per categories of alcohol consumption or binge drinking (95% confidence interval (CI)) as compared to the reference category. Fecundability represents the probability of conceiving within one month. Models were analyzed using Cox proportional hazards models. Fecundability ratios were derived from the Hazard Ratios of the Cox proportional hazards models. Models were adjusted for body mass index, participants age, ethnicity, education level, smoking and drug use. Parity was included in the women's model

Table S5. Associations Of Preconceptional Alcohol Consumption in Women and Men With Fecundability Ratios, Adjusted Model

	Women		Men	
	n	Fecundability ratio (95% CI)	n	Fecundability ratio (95% CI)
**Preconception alcohol use**				
None	127	Reference	74	Reference
<1 glass per week	282	1.22 (0.94, 1.59)	173	1.20 (0.84, 1.73)
≥1 glass per week	417	1.40* (1.07, 1.83)	530	1.30 (0.93, 1.82)
**Preconception binge drinking**				
None	433	Reference	233	Reference
<1 time per month	301	1.22* (1.02, 1.47)	320	1.10 (0.88, 1.38)
≥ 1 time per month	93	1.25 (0.94, 1.66)	227	1.16 (0.90, 1.49)

*P value <0.05. Values represent the fecundability per categories of alcohol consumption or binge drinking (95% confidence interval (CI)) as compared to the reference category. Fecundability represents the probability of conceiving within one month. Models were analyzed using Cox proportional hazards models. Fecundability ratios were derived from the Hazard Ratios of the Cox proportional hazards models. Models were adjusted for body mass index, participants age, ethnicity, education level, smoking and drug use. Parity was included in the women's model

Table S6. Associations of Preconceptional Alcohol Consumption in Women and Men with Fecundability Ratios, Adjusted Model Excluding the Top 5% of Time to Pregnancy

		Women		Men
	n	Fecundability ratio (95% CI)	n	Fecundability ratio (95% CI)
**Preconception alcohol use**				
None	118	Reference	69	Reference
<1 glass per week	265	1.27 (0.97, 1.67)	164	1.16 (0.81, 1.66)
≥1 glass per week	401	1.49* (1.13, 1.95)	512	1.25 (0.90, 1.75)
**Preconception binge drinking**				
None	404	Reference	221	Reference
<1 per month	290	1.23* (1.02, 1.48)	309	1.13 (0.90, 1.41)
≥ 1 time per month	91	1.21 (0.91, 1.60)	220	1.21 (0.94, 1.55)

*P value <0.05. Values represent the fecundability per categories of alcohol consumption or binge drinking (95% confidence interval (CI)) as compared to the reference category. Fecundability represents the probability of conceiving within one month. Models were analyzed using Cox proportional hazards models. Fecundability ratios were derived from the Hazard Ratios of the Cox proportional hazards models. Models were adjusted for body mass index, participants age, ethnicity, education level, smoking and drug use. Parity was included in the women's model

Table S7. Associations Of Preconceptional Alcohol Consumption in Women and Men with Fecundability Ratios, Adjusted Model Including Only First Episodes

	Women		Men	
	n	Fecundability ratio (95% CI)	n	Fecundability ratio (95% CI)
**Preconception alcohol use**				
None	116	Reference	68	Reference
<1 glass per week	269	1.20 (0.91, 1.58)	167	1.17 (0.81, 1.70)
≥1 glass per week	407	1.42* (1.08, 1.87)	509	1.30 (0.92, 1.84)
**Preconception binge drinking**				
None	411	Reference	222	Reference
<1 per month	292	1.24* (1.02, 1.49)	307	1.12 (0.89, 1.42)
≥ 1 time per month	90	1.24 (0.93, 1.65)	219	1.19 (0.92, 1.54)

*P value <0.05. Values represent the fecundability per categories of alcohol consumption or binge drinking (95% confidence interval (CI)) as compared to the reference category. Fecundability represents the probability of conceiving within one month. Models were analyzed using Cox proportional hazards models. Fecundability ratios were derived from the Hazard Ratios of the Cox proportional hazards models. Models were adjusted for body mass index, participants age, ethnicity, education level, smoking and drug use. Parity was included in the women's model

Table S8. Associations Of Preconceptional Alcohol Consumption in Women and Men with Fecundability Ratios, Adjusted Model, Alcohol Consumption 3 Months Preconception.

	Women		Men	
	n	Fecundability ratio (95% CI)	n	Fecundability ratio (95% CI)
**Preconception alcohol use**				
None	460	Reference	286	Reference
<1 glass per week	672	1.12 (0.99, 1.27)	435	0.96 (0.83, 1.12)
1-6 glass per week	1012	1.27* (1.13, 1.43)	1132	0.92 (0.81, 1.06)
6+ glasses per week	120	1.45* (1.18, 1.80)	308	1.00 (0.84, 1.19)
**Preconception binge drinking**				
None	-	-	700	Reference
<1 time per month	-	-	510	1.04 (0.93, 1.17)
≥ 1 time per month, but <1 time per week	-	-	275	1.19* (1.03, 1.38)
≥ 1 time per week	-	-	119	0.93 (0.76, 1.13)

*P value <0.05. Values represent the fecundability per categories of alcohol consumption or binge drinking (95% confidence interval (CI)) as compared to the reference category. Fecundability represents the probability of conceiving within one month. Models were analyzed using Cox proportional hazards models. Fecundability ratios were derived from the Hazard Ratios of the Cox proportional hazards models. Models were adjusted for body mass index, participants age, ethnicity, education level, smoking and drug use. Parity was included in the women's model

Table S9. Associations Of Preconceptional Alcohol Consumption in Women and Men with Fecundability Ratios, Adjusted Model, Alcohol Consumption as The Reference Group

	Women		Men	
	n	Fecundability ratio (95% CI)	n	Fecundability ratio (95% CI)
**Preconception alcohol use**				
None	127	0.82 (0.63, 1.07)	74	0.83 (0.58, 1.19)
<1 glass per week	282	Reference	173	Reference
≥1 glass per week	417	1.15 (0.95, 1.39)	530	1.08 (0.87, 1.35)
**Preconception binge drinking**				
None	433	0.76 (0.64, 0.90)	233	0.82 (0.68, 0.98)*
<1 time per month	301	Reference	320	Reference
≥ 1 time per month	93	0.97 (0.74, 1.26)	227	1.03 (0.78, 1.36)

*P value <0.05. Values represent the fecundability per categories of alcohol consumption or binge drinking (95% confidence interval (CI)) as compared to the reference category. Fecundability represents the probability of conceiving within one month. Models were analyzed using Cox proportional hazards models. Fecundability ratios were derived from the Hazard Ratios of the Cox proportional hazards models. Models were adjusted for body mass index, participants age, ethnicity, education level, smoking and drug use. Parity was included in the women's model

Table S10. Associations of Preconceptional Alcohol Consumption with the Odds of Infertility, Basic Model.

	Women		Men	
	n	Odds ratio (95% CI)	n	Odds ratio (95% CI)
**Preconception alcohol use**				
None	141	Reference	78	Reference
<1 glass per week	322	0.55* (0.37, 0.84)	188	0.59 (0.34, 1.03)
≥1 glass per week	446	0.42* (0.28, 0.62)	583	0.41* (0.25, 0.68)
**Preconception binge drinking**				
None	487	Reference	257	Reference
< 1 time per month	327	0.67* (0.50, 0.88)	346	0.61* (0.44, 0.85)
≥ 1 time per month	96	0.76 (0.49, 1.19)	247	0.52* (0.36, 0.74)

*P value <0.05. Values represent the odds of infertility per categories of alcohol consumption or binge drinking (95% confidence interval (CI)) from logistic regression models, as compared to the reference category.

Table S11. Associations Of Preconceptional Alcohol Consumption in Women and Men with Odds of Infertility, Adjusted Model, Excluding Couples Undergoing Assisted Reproductive Technology

	Women		Men	
	n	Odds ratio (95% CI)	n	Odds ratio (95% CI)
**Preconception alcohol use**				
None	126	Reference	72	Reference
<1 glass per week	274	0.54* (0.34, 0.87)	169	0.64 (0.35, 1.20)
≥1 glass per week	393	0.50* (0.31, 0.80)	505	0.55* (0.31, 0.98)
**Preconception binge drinking**				
None	424	Reference	227	Reference
<1 time per month	280	0.70* (0.50, 0.99)	300	0.81 (0.54, 1.20)
≥ 1 time per month	90	1.01 (0.61, 1.69)	220	0.80 (0.51, 1.26)

*P value <0.05. Values represent the odds of infertility per categories of alcohol consumption or binge drinking (95% confidence interval (CI)) from logistic regression models, as compared to the reference category. Models were adjusted for body mass index, participants age, ethnicity, education level, smoking and drug use. Parity was included in the women's model.

Table S12. Associations Of Alcohol Consumption in Women and Men with Odds of Infertility, Adjusted Model Including Only First Episodes

	Women		Men	
	n	Odds ratio (95% CI)	n	Odds ratio (95% CI)
**Preconception alcohol use**				
None	130	Reference	72	Reference
<1 glass per week	308	0.68 (0.43, 1.06)	181	0.64 (0.35, 1.17)
≥1 glass per week	435	0.57* (0.36, 0.89)	561	0.57 (0.32, 1.00)
**Preconception binge drinking**				
None	464	Reference	245	Reference
<1 per month	318	0.76 (0.55, 1.04)	333	0.78 (0.54, 1.12)
≥ 1 time per month	92	0.82 (0.50, 1.34)	238	0.72 (0.48, 1.09)

*P value <0.05. Values represent the odds of infertility per categories of alcohol consumption or binge drinking (95% confidence interval (CI)) from logistic regression models, as compared to the reference category. Models were adjusted for body mass index, participants age, ethnicity, education level, smoking and drug use. Parity was included in the women's model.

Table S13. Associations Of Alcohol Consumption in Women and Men with Odds of Infertility, Adjusted Model Excluding Top 5% of Time to Pregnancy

	Women		Men	
	n	Odds ratio (95% CI)	n	Odds ratio (95% CI)
**Preconception alcohol use**				
None	133	Reference	74	Reference
<1 glass per week	302	0.58* (0.37, 0.91)	179	0.65 (0.36, 1.18)
≥1 glass per week	429	0.52* (0.33, 0.81)	559	0.57* (0.33, 0.99)
**Preconception binge drinking**				
None	455	Reference	243	Reference
<1 per month	314	0.77 (0.56, 1.06)	333	0.79 (0.55, 1.14)
≥ 1 time per month	95	0.87 (0.54, 1.41)	239	0.71 (0.47, 1.07)

*P value <0.05. Values represent the odds of infertility per categories of alcohol consumption or binge drinking (95% confidence interval (CI)) from logistic regression models, as compared to the reference category. Models were adjusted for body mass index, participants age, ethnicity, education level, smoking and drug use. Parity was included in the women's model.

Table S14. Associations Of Preconceptional Alcohol Consumption in Women and Men With Odds of Infertility, Adjusted Model Including Alcohol Consumption Of Partner

	Women		Men	
	n	Odds ratio (95% CI)	n	Odds ratio (95% CI)
**Preconception alcohol use**				
None	141	Reference	77	Reference
<1 glass per week	322	0.59* (0.37, 0.93)	186	0.80 (0.44, 1.47)
≥1 glass per week	446	0.58* (0.35, 0.95)	582	0.79 (0.44, 1.43)
**Preconception binge drinking**				
None	456	Reference	256	Reference
<1 per month	302	0.79 (0.56, 1.11)	346	0.84 (0.58, 1.22)
≥ 1 time per month	89	1.09 (0.63, 1.88)	245	0.72 (0.46, 1.12)

*P value <0.05. Values represent the odds of infertility per categories of alcohol consumption or binge drinking (95% confidence interval (CI)) from logistic regression models, as compared to the reference category. Models were adjusted for body mass index, participants age, ethnicity, education level, smoking and drug use. Parity was included in the women's model.

Table S15. Associations of Preconceptional Alcohol Consumption in Women and Men with Odds of Infertility, Adjusted Model, 3 months preconception

	Women		Men	
	n	Odds ratio (95% CI)	n	Odds ratio (95% CI)
**Preconception alcohol use**				
None	551	Reference	319	Reference
<1 glass per week	772	0.67* (0.52, 0.85)	490	1.13 (0.81, 1.57)
1-6 glass per week	1095	0.45* (0.35, 0.58)	1240	1.04 (0.77, 1.41)
6+ glasses per week	124	0.34* (0.21, 0.56)	338	0.91 (0.62, 1.33)
**Preconception binge drinking**				
None	-	-	766	Reference
<1 time per month	-	-	549	0.83 (0.63, 1.08)
≥ 1 time per month, but <1 time per week	-	-	292	0.57* (0.39, 0.82)
≥ 1 time per week	-	-	125	0.94 (0.60, 1.47)

*P value <0.05. Values represent the odds of infertility per categories of alcohol consumption or binge drinking (95% confidence interval (CI)) from logistic regression models, as compared to the reference category. Models were adjusted for body mass index, participants age, ethnicity, education level, smoking and drug use. Parity was included in the women's model.

Table S16. Associations of Preconceptional Alcohol Consumption with the Odds of Infertility, Adjusted Model, Alcohol Consumption as The Reference Group

	Women		Men	
	n	Odds ratio (95% CI)	n	Odds ratio (95% CI)
**Preconception alcohol use**				
None	141	0.82 (0.63, 1.07)	78	0.83 (0.58, 1.19)
<1 glass per week	322	Reference	188	Reference
≥1 glass per week	446	1.15 (0.95, 1.39)	583	1.08 (0.87, 1.35)
**Preconception binge drinking**				
None	487	0.82 (0.68, 0.98)*	257	0.91 (0.73, 1.14)
< 1 time per month	327	Reference	346	Reference
≥ 1 time per month	96	1.03 (0.78, 1.36)	247	1.06 (0.86, 1.30)

*P value <0.05. Values represent the odds of infertility per categories of alcohol consumption or binge drinking (95% confidence interval (CI)) from logistic regression models, as compared to the reference category. Models were adjusted for body mass index, participants age, ethnicity, education level, smoking and drug use. Parity was included in the women's model.

Table S17. Associations Of Early-Pregnancy Alcohol Consumption in Women and Men with Hazard Ratios of Miscarriage, Basic Model

	Women		Men	
	n	Hazard ratio (95% CI)	n	Hazard ratio (95% CI)
**Pregnancy alcohol use**				
None	1926	Reference	289	Reference
<1 glass per week	263	1.99* (1.40-2.84)	473	1.20 (0.74-1.95)
≥1 glass per week	208	2.44* (1.70-3.50)	-	
1-6 glasses per week	-	-	1181	1.00 (0.64-1.55)
>6 glasses per week	-	-	323	1.14 (0.67-1.93)
**Pregnancy binge drinking**				
No, binge drinking	1270	Reference	795	Reference
Yes, binge drinking	287	1.69* (1.20-2.39)	-	-
<1 per month	-	-	725	1.23 (0.89-1.70)
≥ 1 time per month, but <1 per week	-	-	408	1.00 (0.67-1.50)
≥1 time per week	-	-	143	0.70 (0.35-1.41)

*P value <0.05. Values represent the Hazard Ratio of miscarriage per categories of alcohol consumption or binge drinking (95% confidence interval (CI)) as compared to the reference category. Models were analyzed using Cox proportional hazards models. Models were adjusted for body mass index, participants age, ethnicity, education level, smoking and drug use. Parity and history of miscarriage were included in the women's model.

Table S18. Associations Of Early-Pregnancy Alcohol Consumption in Women and Men with Hazard Ratios of Miscarriage, Adjusted Model

	Women		Men	
	n	Hazard ratio (95% CI)	n	Hazard ratio (95% CI)
**Pregnancy alcohol use**				
None	1926	Reference	289	Reference
<1 glass per week	263	1.96* (1.37-2.80)	473	1.18 (0.72-1.92)
≥1 glass per week	208	2.40* (1.65-3.49)	-	-
1-6 glasses per week	-	-	1181	0.92 (0.58-1.47)
>6 glasses per week	-	-	323	0.99 (0.57-1.73)
**Pregnancy binge drinking**				
No, binge drinking	1270	Reference	795	Reference
Yes, binge drinking	287	1.53* (1.07-2.20)	-	-
<1 per month	-	-	725	1.19 (0.85-1.68)
≥ 1 time per month, but <1 per week	-	-	408	1.0 (0.65-1.53)
≥1 time per week	-	-	143	0.67 (0.33-1.36)

*P value <0.05. Values represent the Hazard Ratio of miscarriage per categories of alcohol consumption or binge drinking (95% confidence interval (CI)) as compared to the reference category. Models were analyzed using Cox proportional hazards models. Models were adjusted for body mass index, participants age, ethnicity, education level, smoking and drug use. Parity and history of miscarriage were included in the women's model.

Table S19. Associations Of Early-Pregnancy Alcohol Consumption in Women and Men with Hazard Ratios of Miscarriage, Adjusted Model Excluding Couples Undergoing Assisted Reproductive Technology

	Women		Men	
	n	Hazard ratio (95% CI)	n	Hazard ratio (95% CI)
**Pregnancy alcohol use**				
None	1701	Reference	264	Reference
<1 glass per week	247	2.07* (1.43-3.01)	421	1.13 (0.69-1.87)
≥1 glass per week	196	2.55* (1.73-3.77)	-	-
1-6 glasses per week	-	-	1071	0.87 (0.54-1.40)
>6 glasses per week	-	-	296	0.85 (0.47-1.53)
**Pregnancy binge drinking**				
No, binge drinking	1130	Reference	719	Reference
Yes, binge drinking	256	1.58* (1.08-2.33)	-	-
<1 per month	-	-	652	1.19 (0.84-1.70)
≥ 1 time per month, but <1 per week	-	-	376	0.93 (0.59-1.46)
≥1 time per week	-	-	134	0.53 (0.24-1.18)

*P value <0.05. Values represent the Hazard Ratio of miscarriage per categories of alcohol consumption or binge drinking (95% confidence interval (CI)) as compared to the reference category. Models were analyzed using Cox proportional hazards models. Models were adjusted for body mass index, participants age, ethnicity, education level, smoking and drug use. Parity and history of miscarriage were included in the women's model.

Table S20. Associations Of Early-Pregnancy Alcohol Consumption in Women and Men with Hazard Ratios of Miscarriage, Adjusted Model Including Only First Episodes

	Women		Men	
	n	Hazard ratio (95% CI)	n	Hazard ratio (95% CI)
**Pregnancy alcohol use**				
None	1738	Reference	270	Reference
<1 glass per week	241	2.12* (1.46-3.07)	431	1.17 (0.71-1.94)
≥1 glass per week	198	2.63* (1.80-3.84)	-	-
1-6 glasses per week	-	-	1073	0.90 (0.56-1.45)
>6 glasses per week	-	-	299	1.01 (0.57-1.79)
**Pregnancy binge drinking**				
No, binge drinking	1128	Reference	774	Reference
Yes, binge drinking	278	1.46* (1.01-2.11)	-	-
<1 per month	-	-	708	1.19 (0.84-1.68)
≥ 1 time per month, but <1 per week	-	-	397	0.97 (0.63-1.50)
≥1 time per week	-	-	142	0.67 (0.33-1.37)

*P value <0.05. Values represent the Hazard Ratio of miscarriage per categories of alcohol consumption or binge drinking (95% confidence interval (CI)) as compared to the reference category. Models were analyzed using Cox proportional hazards models. Models were adjusted for body mass index, participants age, ethnicity, education level, smoking and drug use. Parity and history of miscarriage were included in the women's model.

Table S21. Associations Of Early-Pregnancy Alcohol Consumption in Women and Men with Hazard Ratios of Miscarriage, Adjusted Model Including Alcohol Consumption of Partner

	Women		Men	
	n	Hazard ratio (95% CI)	n	Hazard ratio (95% CI)
**Pregnancy alcohol use**				
None	1813	Reference	288	Reference
<1 glass per week	249	2.15* (1.45-3.19)	470	1.08 (0.65-1.79)
≥1 glass per week	196	2.91* (1.94-4.37)	-	-
1-6 glasses per week	-	-	1177	0.71 (0.43-1.15)
>6 glasses per week	-		323	0.79 (0.44-1.41)
**Pregnancy binge drinking**				
No, binge drinking	1049	Reference	527	Reference
Yes, binge drinking	264	1.54* (1.01-2.34)	-	-
<1 per month	-	-	444	1.15 (0.76-1.74)
≥ 1 time per month, but <1 per week	-	-	254	0.83 (0.48-1.43)
≥1 time per week	-	-	88	0.41 (0.16-1.08)

*P value <0.05. Values represent the Hazard Ratio of miscarriage per categories of alcohol consumption or binge drinking (95% confidence interval (CI)) as compared to the reference category. Models were analyzed using Cox proportional hazards models. Models were adjusted for body mass index, participants age, ethnicity, education level, smoking and drug use. Parity and history of miscarriage were included in the women's model.

Table S22. Associations Of Early-Pregnancy Alcohol Consumption in Women and Men with Hazard Ratios of Miscarriage, Adjusted Model, 3 months preconception

	Women		Men	
	n	Hazard ratio (95% CI)	n	Hazard ratio (95% CI)
**Pregnancy alcohol use**				
None	481	Reference	289	Reference
<1 glass per week	676	1.21 (0.78-1.88)	473	1.18 (0.72-1.92)
1-6 glasses per week	949	0.77 (0.49-1.22)	1181	0.92 (0.58-1.47)
>6 glasses per week	110	0.86 (0.38-1.93)	323	0.99 (0.57-1.73)
**Pregnancy binge drinking**				
No, binge drinking	-	-	795	Reference
<1 per month	-	-	725	1.19 (0.85-1.68)
≥ 1 time per month, but <1 per week	-	-	408	2.0 (0.65-1.53)
≥1 time per week	-	-	143	0.67 (0.33-1.36)

*P value <0.05. Values represent the Hazard Ratio of miscarriage per categories of alcohol consumption or binge drinking (95% confidence interval (CI)) as compared to the reference category. Models were analyzed using Cox proportional hazards models. Models were adjusted for body mass index, participants age, ethnicity, education level, smoking and drug use. Parity and history of miscarriage were included in the women's model.

Table S23. Associations Of Early-Pregnancy Alcohol Consumption in Women and Men with Hazard Ratios of Miscarriage, Adjusted Model, Alcohol Consumption as The Reference Group

	Women		Men	
	n	Hazard ratio (95% CI)	n	Hazard ratio (95% CI)
**Pregnancy alcohol use**				
None	1926	0.51 (0.36, 0.73)*	289	0.85 (0.52, 1.38)
<1 glass per week	263	Reference	473	Reference
≥1 glass per week	208	1.22 (0.78, 1.93)	-	-
1-6 glasses per week	-	-	1181	0.78 (0.55, 1.12)
>6 glasses per week	-	-	323	0.84 (0.53, 1.34)
**Pregnancy binge drinking**				
No, binge drinking	1270	0.65 (0.45, 0.94)*	795	
Yes, binge drinking	287	Reference	-	0.84 (0.60, 1.18)
<1 per month	-	-	725	Reference
≥ 1 time per month, but <1 per week	-	-	408	0.84 (0.56, 1.25)
≥1 time per week	-	-	143	0.56 (0.28, 1.13)

*P value <0.05. Values represent the Hazard Ratio of miscarriage per categories of alcohol consumption or binge drinking (95% confidence interval (CI)) as compared to the reference category. Models were analyzed using Cox proportional hazards models. Models were adjusted for body mass index, participants age, ethnicity, education level, smoking and drug use. Parity and history of miscarriage were included in the women's model.

Table S24. Associations Of Early-Pregnancy Alcohol Consumption in Women and Men with Odds of Miscarriage, Basic Model

	Women		Men	
	n	Odds ratio (95% CI)	n	Odds ratio (95% CI)
**Pregnancy alcohol use**				
None	1926	Reference	290	Reference
<1 glass per week	263	2.06* (1.42, 3.00)	474	1.20 (0.73, 1.97)
≥1 glass per week	208	2.51* (1.70, 3.72)	-	-
1-6 glasses per week	-	-	1183	0.97 (0.62, 1.52)
>6 glasses per week	-	-	323	1.12 (0.65, 1.92)
**Pregnancy binge drinking**				
No, binge drinking	1270	Reference	798	Reference
Yes, binge drinking	287	1.79* (1.24, 2.58)	-	-
<1 per month	-	-	725	1.20 (0.85, 1.68)
≥ 1 time per month, but <1 per week	-	-	408	0.98 (0.64, 1.48)
≥1 time per week	-	-	143	0.64 (0.31, 1.34

*P value <0.05. Values represent the odds of miscarriage per categories of alcohol consumption or binge drinking (95% confidence interval (CI)) from logistic regression models, as compared to the reference category.

Table S25. Associations Of Early-Pregnancy Alcohol Consumption in Women and Men with Odds of Miscarriage, Adjusted Model Excluding Couples Undergoing Assisted Reproductive Technology

	Women		Men	
	n	Odds ratio (95% CI)	n	Odds ratio (95% CI)
**Pregnancy alcohol use**				
None	1701	Reference	265	Reference
<1 glass per week	249	2.13* (1.43, 3.16)	422	1.13 (0.67, 1.90)
≥1 glass per week	196	2.55* (1.67, 3.90)	-	-
1-6 glasses per week	-	-	1073	0.84 (0.51, 1.37)
>6 glasses per week	-	-	296	0.81 (0.44, 1.49)
**Pregnancy binge drinking**				
No, binge drinking	1131	Reference	722	Reference
Yes, binge drinking	257	1.57* (1.04, 2.38)	-	-
<1 per month	-	-	652	1.16 (0.80, 1.68)
≥ 1 time per month, but <1 per week	-	-	376	0.88 (0.55, 1.41)
≥1 time per week	-	-	134	0.49 (0.21, 1.11)

*P value <0.05. Values represent the odds of miscarriage per categories of alcohol consumption or binge drinking (95% confidence interval (CI)) from logistic regression models, as compared to the reference category. Models were adjusted for body mass index, participants age, ethnicity, education level, smoking and drug use. Parity and history of miscarriage were included in the women’s model.

Table S26. Associations Of Early-Pregnancy Alcohol Consumption in Women and Men with Odds of Miscarriage, Adjusted Model Including Only First Episodes

	Women		Men	
	n	Odds ratio (95% CI)	n	Odds ratio (95% CI)
**Pregnancy alcohol use**				
None	1739	Reference	271	Reference
<1 glass per week	243	2.20* (1.48, 3.27)	432	1.16 (0.69, 1.96)
≥1 glass per week	198	2.65* (1.75, 4.02)	-	-
1-6 glasses per week	-	-	1074	0.84 (0.51, 1.38)
>6 glasses per week	-	-	299	0.96 (0.53, 1.75)
**Pregnancy binge drinking**				
No, binge drinking	1129	Reference	777	Reference
Yes, binge drinking	279	1.48 (1.00, 2.19)	-	-
<1 per month	-	-	708	1.16 (0.81, 1.66)
≥ 1 time per month, but <1 per week	-	-	397	0.93 (0.59, 1.47)
≥1 time per week	-	-	142	0.63 (0.30, 1.33)

*P value <0.05. Values represent the odds of miscarriage per categories of alcohol consumption or binge drinking (95% confidence interval (CI)) from logistic regression models, as compared to the reference category. Models were adjusted for body mass index, participants age, ethnicity, education level, smoking and drug use. Parity and history of miscarriage were included in the women's model.

Table S27. Associations Of Early-Pregnancy Alcohol Consumption in Women and Men with Odds of Miscarriage, Adjusted Model Including Alcohol Consumption of Partner

	Women		Men	
	n	Odds ratio (95% CI)	n	Odds ratio (95% CI)
**Pregnancy alcohol use**				
None	1815	Reference	289	Reference
<1 glass per week	251	2.01* (1.34, 3.01)	471	1.09 (0.66, 1.82)
≥1 glass per week	196	2.67* (1.73, 4.12)	-	-
1-6 glasses per week	-	-	1179	0.72 (0.44, 1.18)
>6 glasses per week	-	-	323	0.77 (0.42, 1.39)
**Pregnancy binge drinking**				
No, binge drinking	1050	Reference	529	Reference
Yes, binge drinking	265	1.59* (1.02, 2.47)	-	-
<1 per month	-	-	444	1.13 (0.73, 1.75)
≥ 1 time per month, but <1 per week	-	-	254	0.83 (0.47, 1.46)
≥1 time per week	-	-	88	0.38 (0.14, 1.04)

*P value <0.05. Values represent the odds of miscarriage per categories of alcohol consumption or binge drinking (95% confidence interval (CI)) from logistic regression models, as compared to the reference category. Models were adjusted for body mass index, participants age, ethnicity, education level, smoking and drug use. Parity and history of miscarriage were included in the women's model.

Table S28. Associations Of Early-Pregnancy Alcohol Consumption in Women and Men with Odds of Miscarriage, adjusted model, 3 months preconception

	Women		Men	
	n	Odds ratio (95% CI)	n	Odds ratio (95% CI)
**Pregnancy alcohol use**				
None	481	Reference	290	Reference
<1 glass per week	678	1.27 (0.81, 2.00)	474	1.17 (0.70, 1.94)
1-6 glasses per week	950	0.77 (0.48, 1.24)	1183	0.88 (0.55, 1.43)
>6 glasses per week	110	0.85 (0.37, 1.97)	323	0.95 (0.53, 1.70)
**Pregnancy binge drinking**				
No, binge drinking	-	-	798	Reference
<1 per month	-	-	725	1.16 (0.81, 1.66)
≥ 1 time per month, but <1 per week	-	-	408	0.96 (0.62, 1.50)
≥1 time per week	-	-	143	0.63 (0.30, 1.32)

*P value <0.05. Values represent the odds of miscarriage per categories of alcohol consumption or binge drinking (95% confidence interval (CI)) from logistic regression models, as compared to the reference category. Models were adjusted for body mass index, participants age, ethnicity, education level, smoking and drug use. Parity and history of miscarriage were included in the women's model.

Table S29. Associations Of Early-Pregnancy Alcohol Consumption in Women and Men with Odds of Miscarriage, Adjusted Model, Alcohol Consumption as The Reference Group

	Women		Men	
	n	Odds ratio (95% CI)	n	Odds ratio (95% CI)
**Pregnancy alcohol use**				
None	1926	0.48 (0.33, 0.70)*	290	0.80 (0.49, 1.33)
<1 glass per week	263	Reference	474	Reference
≥1 glass per week	208	1.18 (0.72, 1.93)	-	-
1-6 glasses per week	-	-	1183	0.75 (0.52, 1.08)
>6 glasses per week	-	-	323	0.86 (0.53, 1.39)
**Pregnancy binge drinking**				
No, binge drinking	1270	0.60 (0.41, 0.88)*	798	0.83 (0.58, 1.18)
Yes, binge drinking	287	Reference	-	-
<1 per month	-	-	725	Reference
≥ 1 time per month, but <1 per week	-	-	408	0.85 (0.56, 1.29)
≥1 time per week	-	-	143	0.55 (0.27, 1.13)

*P value <0.05. Values represent the odds of miscarriage per categories of alcohol consumption or binge drinking (95% confidence interval (CI)) from logistic regression models, as compared to the reference category. Models were adjusted for body mass index, participants age, ethnicity, education level, smoking and drug use. Parity and history of miscarriage were included in the women's model.

STROBE Statement—checklist of items that should be included in reports of observational studies

	Item No.	Recommendation	Page No.	Relevant text from manuscript
**Title and abstract**	1	(*a*) Indicate the study's design with a commonly used term in the title or the abstract	1	In a population-based cohort study from preconception onwards among 3,225 women and their partners.
		(*b*) Provide in the abstract an informative and balanced summary of what was done and what was found	1	We examined the associations of preconception and early-pregnancy alcohol consumption, assessed by questionnaires, with fecundability (per-cycle probability of conceiving), infertility (time to pregnancy >12 months or use of assisted reproductive technology), and miscarriage (pregnancy loss before 22 weeks of gestation). As compared to no alcohol consumption, preconception intake of <1 glass/week and ≥1

				glass/week among women was associated with lower odds of infertility (Odds Ratio (OR) 0.61, 95% Confidence Interval (CI) 0.39, 0.94, and OR 0.54, 95% CI 0.35, 0.84, respectively). As compared to no alcohol consumption, early-pregnancy alcohol consumption among women of <1 glass/week and ≥1 glass/week was associated with increased odds of miscarriage (OR 1.99, 95% CI 1.36, 2.92 and OR 2.37, 95%CI 1.58, 3.57). Early-pregnancy binge drinking among women was also associated with increased odds of miscarriage (OR 1.52, 95%CI 1.04, 2.24). Alcohol consumption among men was not associated with infertility or miscarriage risks.
**Introduction**				
Background/rationale	2	Explain the scientific background and rationale for the investigation being reported	2	In the Netherlands, approximately 50% of the women report mild to

				moderate alcohol consumption just before or during early pregnancy. Early pregnancy seems to be a critical exposure window for the effects of alcohol on embryonic, fetal, and childhood outcomes. However, the effects of mild-to-moderate alcohol consumption on fertility and early pregnancy outcomes are not well-known.
Objectives	3	State specific objectives, including any prespecified hypotheses	2	We hypothesized that already mild to moderate periconceptional alcohol consumption among both women and men leads to a higher risk of infertility and miscarriage. In a population-based cohort study from preconception onwards among 3,225 women and their partners, we examined the associations of alcohol consumption with fecundability and risks of infertility and miscarriage, considering various socio-

				demographic, lifestyle-related, and pregnancy-related factors.
**Methods**				
Study design	4	Present key elements of study design early in the paper	2	This study was embedded in the Generation R *Next* Study, a population-based prospective cohort study in Rotterdam, the Netherlands, from the preconception period onwards.
Setting	5	Describe the setting, locations, and relevant dates, including periods of recruitment, exposure, follow-up, and data collection	2	This study was embedded in the Generation R *Next* Study, a population-based prospective cohort study in Rotterdam, the Netherlands, from the preconception period onwards. Women and their partners could participate if they were 18 years or older, actively trying to conceive, or pregnant and living in Rotterdam. Participants were enrolled from August 9, 2017, to July 1, 2021. Enrollment was intended for those in the preconception or

				early-pregnancy period but was permitted until delivery.
Participants	6	(*a*) *Cohort study*—Give the eligibility criteria, and the sources and methods of selection of participants. Describe methods of follow-up<br><br>*Case-control study*—Give the eligibility criteria, and the sources and methods of case ascertainment and control selection. Give the rationale for the choice of cases and controls<br><br>*Cross-sectional study*—Give the eligibility criteria, and the sources and methods of selection of participants	3	Information on alcohol consumption was obtained using questionnaires administered during the preconception period and the first trimester.<br><br>Time to pregnancy and mode of conception were assessed through preconception and early-pregnancy questionnaires, as described previously.
		(*b*) *Cohort study*—For matched studies, give matching criteria and number of exposed and unexposed<br><br>*Case-control study*—For matched studies, give matching criteria and the number of controls per case	-	-
Variables	7	Clearly define all outcomes, exposures, predictors, potential confounders, and effect modifiers. Give diagnostic criteria, if applicable	3-5	**Methods:** Preconception and early-pregnancy alcohol consumption in women and men<br><br>**Methods:** Time to Pregnancy and Miscarriage<br><br>**Methods:** Covariates

Data sources/ measurement	8*	For each variable of interest, give sources of data and details of methods of assessment (measurement). Describe comparability of assessment methods if there is more than one group	3	Information on alcohol consumption was obtained using questionnaires administered during the preconception period and the first trimester.<br><br>Time to pregnancy and mode of conception were assessed through preconception and early-pregnancy questionnaires, as described previously.
Bias	9	Describe any efforts to address potential sources of bias	4	First, baseline characteristics were stratified by alcohol consumption categories, and differences between groups were assessed using the Independent Student T-test, Mann-Whitney U tests, and χ^2 tests for categorical variables. Second, we performed a non-response analysis by comparing characteristics of women and men with and without information on time to pregnancy or miscarriage using the Independent Student T-test, Mann-

				Whitney U tests, and χ^2 tests for categorical variables
Study size	10	Explain how the study size was arrived at	Supplement	Figure S1: flowchart of the study population
Quantitative variables	11	Explain how quantitative variables were handled in the analyses. If applicable, describe which groupings were chosen and why	3	Preconception alcohol intake among women and men was categorized as none, <1 glass per week, or ≥1 glass per week. Preconception binge drinking, defined as consuming ≥6 glasses of alcohol on one day, was categorized as no binge drinking, <1 time per month, and ≥1 time per month. Due to both the limited number of preconceptionally participating women and their low alcohol intake, further subdivision of the higher intake category was not feasible.<br><br>Women were asked to report their average alcohol consumption during the previous three months. If these data were missing, alcohol consumption prior to

conception was used, either from the three months before conception or, if unavailable, the average intake during the 12 months prior to conception. During early pregnancy, alcohol intake was categorized as none, <1 glass per week, or ≥1 glass per week, whereas binge drinking was categorized as yes or no. During early-pregnancy, men were asked to report their average alcohol consumption in the three months prior to conception. When this information was unavailable, data from the women's questionnaires regarding their partners' drinking habits were used. If this was also unavailable, their alcohol intake for the 12 months prior to conception, reported by the female partner, was used, assuming that the alcohol intake of the partner remains fairly stable over time. Among men, early-pregnancy alcohol intake was categorized as none; <1 glass per week; 1–6 glasses per week; or >6 glasses per week,

				whereas binge drinking was categorized as no; <1 time per month; >1 time per month but less than once per week; ≥1 time per week. Binge drinking by the male partner was only reported by women.<br><br>Fecundability was defined as the per-cycle probability of conceiving, defined as 28 days. Time to pregnancy was categorized into two groups: a time to pregnancy of 12 months or less (fertile) and a time to pregnancy, or in the case of not conceiving, a duration of actively pursuing pregnancy of more than 12 months or using assisted reproductive technology (infertile).
Statistical methods	12	(*a*) Describe all statistical methods, including those used to control for confounding	4	First, baseline characteristics were stratified by alcohol consumption categories, and differences between groups were assessed using independent Student t-tests, Mann-Whitney U tests, and χ2

tests for categorical variables. Second, a non-response analysis was conducted by comparing characteristics of women and men with and without information on time to pregnancy or miscarriage using the Independent Student t-tests, Mann-Whitney U tests, and $\chi 2$ tests for categorical variables. Third, we analyzed the association of preconception alcohol consumption with fecundability among women and men using Cox proportional hazards models (R package survival). The fecundability time-to-event variable was measured in months (28 days) from the start of pregnancy pursuit until conception. In cases where conception was not achieved, time-to-event was measured up to the last reported pregnancy attempt, defined as the earlier of the voluntary withdrawal date and the study end date, and right-censored. Women receiving assisted conception had an uncertain time to

pregnancy and were therefore excluded from the fecundability analyses. The resulting hazard ratio (HR) from the Cox model corresponds to the fecundability ratio (FR) (Methods S1). We examined the associations of early-pregnancy alcohol consumption among women and men with time to miscarriage using Cox proportional hazards models (R package survival). The outcome was the occurrence or non-occurrence of a miscarriage. The time-to-event variable for miscarriage was based on gestational age in weeks, whereas stillbirth or live birth were censored at 22 weeks. Ectopic pregnancies and induced abortions were categorized as no miscarriage because of underlying pathophysiological differences and were treated as censoring events at the corresponding gestational age. For both models, we evaluated the proportional hazards assumption for the

covariates through Schoenfeld residuals and assessed influence using deviance residuals. Fifth, we analyzed the associations of alcohol consumption with the odds of infertility and miscarriage among women and men through logistic regression models. We conducted 7 sensitivity analyses. First, since some women participated multiple times, we repeated the analyses using only their first study participation. Second, we excluded participants who underwent assisted conception to assess whether any association was affected by assisted reproductive technology. Third, to account for the right-skewed distribution of time-to-pregnancy, the Cox proportional hazards regression was repeated after excluding the top 5% of the distribution. Fourth, to account for potential confounding from partners' alcohol consumption, we repeated the analyses with

adjustment for partners' alcohol consumption. Fifth, we repeated the time-to-pregnancy analyses with alcohol consumption in the past three months, to account for any changes in alcohol consumption patterns in the preconception period. Sixth, we repeated the analyses using <1 glass per week and <1 time per month as the reference groups to assess whether the effect estimates change if we use occasional users as the reference group. Seventh, we repeated the analyses by applying the 3-month preconception alcohol consumption in the miscarriage analyses, to harmonize categories and timeframes across sexes. Missing data on covariates ranged from 0.1% to 41.5% for the partner's BMI. Missing values were imputed using multiple imputations by chained equations (R package mice) to reduce bias. Multiple imputation reliably handles missing data up to 50% under

			the Missing at Random assumption. All models used multiple imputed datasets with Rubin's rules to pool estimates. Analyses were performed in R 4.2.3. P values from two-sided tests, significance at $P < .05$.
	(*b*) Describe any methods used to examine subgroups and interactions	4	First, since some women participated multiple times, we repeated the analyses using only their first study participation. Second, we excluded participants who underwent assisted conception to assess whether any association was affected by assisted reproductive technology. Third, to account for the right-skewed distribution of time-to-pregnancy, the Cox proportional hazards regression was repeated after excluding the top 5% of the distribution. Fourth, to account for potential confounding from partners' alcohol consumption, we repeated the analyses with adjustment for partners' alcohol consumption. Fifth,

			we repeated the time-to-pregnancy analyses with alcohol consumption in the past three months, to account for any changes in alcohol consumption patterns in the preconception period. Sixth, we repeated the analyses using <1 glass per week and <1 time per month as the reference groups to assess whether the effect estimates change if we use occasional users as the reference group. Seventh, we repeated the analyses by applying the 3-month preconception alcohol consumption in the miscarriage analyses, to harmonize categories and timeframes across sexes.
	(*c*) Explain how missing data were addressed	4	Missing values were imputed using multiple imputations by chained equations (R package mice) to reduce bias.
	(*d*) *Cohort study*—If applicable, explain how loss to follow-up was addressed<br>*Case-control study*—If applicable, explain how matching of cases and controls was addressed		

	Cross-sectional study—If applicable, describe analytical methods taking account of sampling strategy	
	(*e*) Describe any sensitivity analyses	First, since some women participated multiple times, we repeated the analyses using only their first study participation. Second, we excluded participants who underwent assisted conception to assess whether any association was affected by assisted reproductive technology. Third, to account for the right-skewed distribution of time-to-pregnancy, the Cox proportional hazards regression was repeated after excluding the top 5% of the distribution. Fourth, to account for potential confounding from partners' alcohol consumption, we repeated the analyses with adjustment for partners' alcohol consumption. Fifth, we repeated the time-to-pregnancy analyses with alcohol consumption in the past three months, to account for any changes in alcohol consumption

				patterns in the preconception period. Sixth, we repeated the analyses using <1 glass per week and <1 time per month as the reference groups to assess whether the effect estimates change if we use occasional users as the reference group. Seventh, we repeated the analyses by applying the 3-month preconception alcohol consumption in the miscarriage analyses, to harmonize categories and timeframes across sexes.
**Results**				
Participants	13*	(a) Report numbers of individuals at each stage of study—eg numbers potentially eligible, examined for eligibility, confirmed eligible, included in the study, completing follow-up, and analysed	Supplement	See figure S1: flowchart of the study population
		(b) Give reasons for non-participation at each stage	Supplement	See figure S1: flowchart of the study population
		(c) Consider use of a flow diagram	Supplement	See figure S1: flowchart of the study population
Descriptive data	14*	(a) Give characteristics of study participants (eg demographic, clinical, social) and information on exposures and potential confounders	5-6	See table 1
		(b) Indicate number of participants with missing data for each variable of interest	Supplement	See table S2 and S3
		(c) *Cohort study*—Summarise follow-up time (eg, average and total amount)	4	Couples were included between August 9, 2017 and

				July 1, 2021. Inclusion was aimed at those in preconception or early-pregnancy but was allowed until delivery.
Outcome data	15*	*Cohort study*—Report numbers of outcome events or summary measures over time	10, 12-13, supplement	Table 2-3, tables S4-S29
		Case-control study—Report numbers in each exposure category, or summary measures of exposure	n/a	n/a
		Cross-sectional study—Report numbers of outcome events or summary measures	n/a	n/a
Main results	16	(*a*) Give unadjusted estimates and, if applicable, confounder-adjusted estimates and their precision (eg, 95% confidence interval). Make clear which confounders were adjusted for and why they were included	23-24, supplement	Table 2-3, tables S4-S23
		(*b*) Report category boundaries when continuous variables were categorized	n/a	n/a
		(*c*) If relevant, consider translating estimates of relative risk into absolute risk for a meaningful time period	n/a	*n/a*

Continued on next page

Other analyses	17	Report other analyses done—eg analyses of subgroups and interactions, and sensitivity analyses	7-9	Methods: statistical analysis
**Discussion**				
Key results	18	Summarise key results with reference to study objectives	5	In this population-based prospective cohort study from the preconception period onwards, we observed that preconceptional low-to-moderate alcohol consumption among women was associated with higher fecundability and lower odds of infertility, whereas alcohol consumption during early-pregnancy was consistently associated with an increased risk of miscarriage. Among men, alcohol consumption was not associated with infertility, and miscarriage.
Limitations	19	Discuss limitations of the study, taking into account sources of potential bias or imprecision. Discuss both direction and magnitude of any potential bias	16	This study has several limitations. First, non-response analyses showed that the included and excluded populations differed, potentially limiting the generalizability of the results to other populations. Excluded participants consumed alcohol, binge drank and used illicit drugs

more frequently during early-pregnancy and were more often multiparous than those included in the analyses. Second, the potential influence of the sick quitter bias must be considered, given that abstainers in our cohort systematically differ from those consuming alcohol. Abstainers had a higher baseline prevalence of prior miscarriage and a longer time to pregnancy. Using this group in our analyses may have underestimated the effect of preconceptional alcohol consumption, falsely suggesting it protects against infertility and increases fecundability. Third, alcohol consumption was self- or partner-reported and assessed via questionnaires, as commonly done in large cohort studies. Recall bias and social desirability bias may have caused misclassification, especially during early-pregnancy. Furthermore, early miscarriages could have been misclassified as non-

		conceptions, especially if the miscarriage occurred before clinical pregnancy recognition. Miscarriages are frequently undiagnosed as they are mistaken for heavy and late menstruation. Potentially, if alcohol consumption increases the risk of unrecognized very early pregnancy loss, it would prolong the time to pregnancy and decrease the observed risk of miscarriage, leading to an overestimation of the association of alcohol with fecundability and infertility and an underestimation of the association of alcohol with miscarriage. Fourth, the questionnaires were collected before the miscarriage diagnosis, and no data collection was performed after the miscarriage diagnosis. However, the miscarriage might have already subclinically occurred before the diagnosis was made by ultrasound or clinical symptoms. Although this effect is expected to be minimal, it could potentially

lead to reversed causality. Furthermore, the time windows for the alcohol assessments do not enable identification of time-specific effects during ovulation, implantation, or early pregnancy. Fifth, because higher alcohol consumption was rare in our study group, dividing it into more categories led to very small group sizes in the higher-intake categories, reducing statistical power. As a result, alcohol consumption was grouped broadly into none, less than one glass per week, and one or more glasses per week for the fertility analyses. This broad categorization combines different drinking habits and limits the ability to analyze detailed dose-response relationships. Given our sample sizes, smaller impacts of male alcohol consumption may remain undetected, indicating a lack of evidence for an association rather than proof of no effect. Finally, due to the observational nature of this cohort study, residual

				confounding cannot be completely excluded. Therefore, the statistical associations should be interpreted with caution, as they do not provide proof of a clinically protective effect of alcohol on reproductive outcomes.
Interpretation	20	Give a cautious overall interpretation of results considering objectives, limitations, multiplicity of analyses, results from similar studies, and other relevant evidence	17	The associations of low-to-moderate preconception alcohol consumption with lower odds of infertility might reflect healthier lifestyles, socioeconomic factors, and residual confounding by health-related abstinence from alcohol, all of which are associated with greater fertility. Therefore, preconception and early pregnancy counseling should mainly focus on the risk of miscarriage and fetal health outcomes.
Generalisability	21	Discuss the generalisability (external validity) of the study results	16	First, non-response analyses showed that the included and excluded populations differed, potentially limiting the

				generalizability of the results to other populations.
**Other information**				
Funding	22	Give the source of funding and the role of the funders for the present study and, if applicable, for the original study on which the present article is based	17	The Generation *R* Study is financially supported by the Erasmus University Medical Centre, Rotterdam, the Erasmus University Rotterdam, and the Netherlands Organization for Health Research and Development. This work was supported by a grant from ZonMW, grant number 05430052110007. Vincent Jaddoe received an European Research Council Consolidator Grant (ERC-2014-CoG-648916, EMBRYOandLATERHEALTH), and European Research Council Advanced Grant (ERC-2025-Adv-10126594, PreconceptionOrigins). R.G. received funding from the Netherlands Organization for Health Research and Development (NWO, ZonMw VIDI 09150172110034, and NWO, ZonMW, grant number 05430052110007), from the Dutch Diabetes Foundation

	(Grant NO. 2024.28.001), and from the European Union (ERC, OBESE-EMBRYO, ERC-2024-STG-101161004), and the Erasmus MC 2 Research Synergy Grant 2024. Views and opinions expressed are, however, those of the author(s) only and do not necessarily reflect those of the European Union or the European Research Council. Neither the European Union nor the granting authority can be held responsible for them.

*Give information separately for cases and controls in case-control studies and, if applicable, for exposed and unexposed groups in cohort and cross-sectional studies.

Note: An Explanation and Elaboration article discusses each checklist item and gives methodological background and published examples of transparent reporting. The STROBE checklist is best used in conjunction with this article (freely available on the Web sites of PLoS Medicine at http://www.plosmedicine.org/, Annals of Internal Medicine at http://www.annals.org/, and Epidemiology at http://www.epidem.com/). Information on the STROBE Initiative is available at www.strobe-statement.org.
